# The evolution and improvement of medical-vocational education policies: a perspective based on the IAD framework

**DOI:** 10.3389/fpubh.2026.1734303

**Published:** 2026-04-01

**Authors:** Xingxin Liu, Jinsi Liu, Jayakaran Mukundan

**Affiliations:** 1 School of Education, Taylor’s University, Subang Jaya, Selangor, Malaysia; 2 Local Government Public Service Innovation Research Center, School of Political Science and Public Administration, Wuhan University, Wuhan, China

**Keywords:** IAD framework, medical-vocational education, policy changes, policy improvement, text mining

## Abstract

With the increasing aging population, society urgently needs a group of experienced medical professionals. However, current medical vocational education faces many difficulties. Only by formulating scientific, medical, and vocational education policies can we effectively promote the modernization of medical and vocational education. Therefore, it is imperative to streamline policy changes. This study focuses on China’s medical-vocational education policy and first divides the policy into four stages. Text analysis methods, such as clustering, examine the key challenges and primary characteristics of policies at various stages of development. Next, the Institutional Analysis and Development (IAD) framework was employed to construct a policy change model and to thoroughly examine the policy change mechanism from the perspectives of natural attributes, community attributes, and application rules. Finally, corresponding countermeasures and suggestions are put forward in response to the shortcomings in developing medical vocational education policies. The research found that the changes in China’s medical-vocational education policy show a progressive evolution path of “natural attributes-community attributes-institutional rules.” The driving force behind the three changes stems from the coordinated evolution of exogenous variables, adjustments in institutional attributes, and changes in actor interaction modes. In essence, it is the dynamic adaptation of the institutional system to changes in the external environment. The government should continually adapt to the evolving needs of the medical industry and effectively support economic development, a crucial requirement for advancing medical-vocational education. At the same time, it should actively promote the integration of industry and education, as well as cooperation between medical schools in medical vocational education, and actively integrate with international medical vocational education.

## 1 Introduction

In the Chinese context, Medical Vocational Education (MVE) refers to an educational and training system designed to cultivate skilled personnel in the medical and healthcare field, primarily targeting secondary and higher vocational education levels (1). It mainly encompasses education and training in nursing, medical technology, pharmaceutical technology, rehabilitation therapy, public health, and related fields. It is an essential component of China’s vocational education system. On the one hand, through systematic theoretical teaching and practical training, it lays a professional foundation for students to work in medical and health services. On the other hand, it supplies society with practical talents needed in areas such as primary healthcare and health services. This system encompasses not only formal schooling but also continuing education and on-the-job training, thereby establishing a key link in the development of a comprehensive, lifelong healthcare talent training system (2). The development of MVE is a dynamic and complex process that involves multiple fields, including national and regional politics, economy, culture, and society. In this complex interaction, only by formulating appropriate medical vocational education policies (MVEP) based on the actual context can their effectiveness and sustainability be ensured (3). Therefore, MVEP must be constantly adjusted and reformed to adapt to the needs of social and technological development. From a global perspective, competition in education is becoming increasingly fierce, and the aging population has put tremendous pressure on the medical industry and the employment market. At the same time, rapid social and technological changes have brought multiple challenges to medical vocational education. For example, the United States continuously adjusts its specialist physician training structure to respond to societal needs through trend analysis and policy forecasting of postgraduate medical education models (4). Australia focuses on strengthening its medical education and research capabilities, guiding policy and teaching quality improvement through cooperation between professional associations and funding agencies (5). Furthermore, Pakistan optimizes medical education governance through the analysis of policy tools (6). Vietnam systematically improves its medical education level by developing key institutional policies such as national competence standards (7).

Currently, the medical education system still faces several problems (8). The most prominent issue is that the quantity and quality of medical talent training cannot meet the growing social needs (9). First, the skills shortage in the medical field remains severe, particularly in training primary medical and healthcare personnel (10). On the one hand, the quantity and quality of professional and technical personnel training cannot keep up with the changes in medical service needs. On the other hand, a significant gap exists between MVE and actual medical service needs, particularly in fundamental medicine and clinical skills training (11). Additionally, negative population growth and the resulting labor shortage have also presented new challenges to MVE. In particular, there is an urgent need to improve the training of technical medical personnel and nursing staff (12). Secondly, the government’s response to the labor market demand of the medical industry is not timely enough, and the financial support is also insufficient (13). The relevant departments have a limited guiding role in determining the curriculum setting and talent training direction of MVE. This has led to the failure of medical education to play its key role in industrial skill innovation fully and the dissemination of technology (14). In addition, the willingness of adults to participate in MVE is generally low due to problems such as insufficient motivation for adult learning and scattered and chaotic training market resources (15). Finally, the distribution of MVE resources is uneven among regions, particularly in economically underdeveloped areas, which face challenges such as inadequate school conditions and insufficient teaching facilities. This affects the quality of medical education and, in turn, the overall improvement of medical services (16).

These problems are not only challenges in the field of medical vocational education in China, but also everyday problems facing the world. Since the reform and opening up, China’s vocational education has made remarkable progress, supported by national policies (17). Especially in medical vocational education, the government has gradually promoted the training of high-quality technical talents needed in the medical industry through policy guidance and resource allocation (1). With the expansion of the school scale and the increase in the employment rate, MVE has gradually achieved the government’s predetermined education policy goals. This has alleviated the employment pressure of young people of school age while meeting the growing demand for medical services. The positioning of medical vocational schools has become increasingly apparent, with training objectives becoming clearer, and a characteristic school-running model has been established to a certain extent. Some schools have formed a staggered competitive relationship through unique professional settings and teaching characteristics (18). The complementary model has promoted the common development of MVE (19). On May 1, 2022, the newly revised “Vocational Education Law of the People’s Republic of China” took effect^1^. The document clearly states that MVE and vocational training are equally important. It also requires strengthening the integration of industry and education, promoting the connection between medical education and actual medical service needs, and forming a modern MVE system for lifelong learning for all people. However, despite the many achievements of the policy, China’s current MVE still faces some challenges and problems. For example, the quality of education in some regions and colleges is uneven, and the depth and breadth of industry-education integration still need to be strengthened. The overall level and internationalization of MVE need to be improved (20).

To address these issues, it is essential to understand the evolution of policy and its context. So, how has China’s vocational education policy evolved? How should it be optimized in the future? To answer these questions, the evolution process must be clarified. This lays a theoretical foundation for optimizing policies and improving educational outcomes (21). Looking back at the historical evolution of China’s MVEP, the government has continuously adjusted and optimized relevant policies in response to the changing needs of the time to meet the demand for medical and health personnel in the new era. To deeply analyze the evolutionary logic of the complex institutional system of medical vocational education policy, this paper adopts the IAD framework as its core analytical tool. Compared to linear policy process models or single-attribute theories, the advantage of the IAD framework lies in its systematic integration of exogenous variables, action scenarios, and interaction patterns (22). It can reveal the mechanism of policy change under the constraints of natural material conditions, community attributes, and dynamic rules. This choice is highly consistent with the research objectives: to move beyond the static description of policy texts and instead explore the structural driving forces and multi-stakeholder interactions behind its changes (23). Therefore, using the IAD framework not only helps to deconstruct the complex driving forces of the changes in China’s medical vocational education policy but also provides an integrated analytical perspective for understanding its future improvement path. The IAD framework offers an effective analytical tool (24) to help understand how MVEP affects different groups and individuals (25) and also reveals which social and economic factors drive policy changes (26). This article will focus on China’s MVEP since the reform and opening up, and it will use text mining methods and the IAD framework to analyze its evolution path in depth. It will also explore ways to improve policies further and provide a reference for future policymaking. The innovation of this paper lies in the following. First, the evolution of China’s MVEP was systematically sorted out. The differences in policies at different stages were compared and analyzed through text-mining methods. Second, the IAD theoretical framework was used to explore the reasons for the changes in MVEP from multiple perspectives (24), including external variables, action scenarios, and interaction patterns. This provides new perspectives and ideas for optimizing policies.

## 2 Literature review

In the process of cultivating medical vocational education, it should be adapted to the requirements of modernization. Students should not only master professional medical skills and techniques but also have comprehensive qualities and practical abilities. In the study of MVE in different countries and regions, scholars have found through the analysis of policy texts that other cultures, historical backgrounds, and socio-economic structures influence the development of MVE in various countries. Factors such as culture, race, and class level in Israel directly affect the policy design of its MVE (27). Germany has developed a social demand-oriented medical education and training model by combining medical institutions, vocational schools, and trade unions, forming a representative “German model” (28). Due to the implementation of the federal system, Australia faces the problem of an overlap between state and federal governments when formulating MVEP. Therefore, it is essential to enhance cooperation and coordination between national and state governments (29). The MVEPs of EU countries vary, reflecting the unique needs of different countries in their historical, cultural, economic, and political backgrounds (15). In the study of the significance of MVE, the Portuguese case demonstrated that a medical education method emphasizing the combination of learning and practice helps achieve short-term training goals (30). Additionally, MVE plays a crucial role in the transition of young people from school to medical careers. In particular, vocational training focused on clinical work can significantly enhance employment opportunities (31). In countries that implement dual medical education, students can achieve a balance between academic studies and practical work, thereby enhancing their clinical practice skills and improving their competitiveness in the job market. This dual system is beneficial for students transitioning from an educational environment to the workforce, effectively enhancing the social benefits of MVE (32). In a study of MVE in China, graduates who received MVE had higher career returns than those who did not receive MVE (33). Driven by China’s targeted poverty alleviation policy, the country has increased its investment in MVE in remote areas. Poor students have access to better educational resources, which provides an effective guarantee for improving educational equity and narrowing regional gaps (34). Educational equity in MVE is reflected in three aspects. First, institutions of different types of MVE should enjoy equal policy and legal status. Institutional discrimination should be eliminated to ensure all educated people have equal educational opportunities. Second, students should select a medical major that aligns with their personal interests and career goals, without being limited by external factors. Ultimately, the talents cultivated by different types of MVE are equally essential and play a crucial role in building a modern medical system. China’s MVE system has gradually improved in these aspects, positively promoting social benefits and public health construction. Against the background of China’s huge population base, the scale and quality of MVE have been continuously improved. Improving rural medical services has brought significant economic and social benefits to society (16).

However, MVE also faces challenges in the development process, including changes in labor market demand and social evaluation. In the social evaluation research of MVE, the development of MVE is also driven by the labor market’s demand. For example, the expansion of higher MVE in Sweden has increased the participation of policy priority groups, but has also led to certain educational inequalities (35). For developing countries, improving MVE is necessary. However, due to insufficient social security and unstable employment, it is challenging to establish a comprehensive and systematic training system for medical professionals (36). Therefore, it is essential to conduct some basic learning before MVE training. This will help improve the level of students, and all this depends on the positioning of MVE in the labor market and education system (37). A survey of MVE practitioners in two Chinese cities found that pre-employment education significantly improved workability and promoted better work performance. However, compared with general higher education, the effect of secondary MVE on improving work performance is relatively limited (38). The universal national vocational qualification policy implemented in England and Wales has become an essential supplement to MVE and has received widespread attention in the early stages. However, this policy has also been questioned due to conflicts between academic and career goals, as well as long-term and short-term needs (39). The performance of graduates in the labor market often depends on their overall performance during their time in school, and the quality of teaching by highly educated teachers can significantly improve students’ employment rates. Additionally, policies to enhance regional labor mobility have positive implications for employment (40).

Implementing MVE has profoundly impacted social medical needs; however, specific challenges remain in the social cognition and policy system. The talents cultivated by MVE can better meet society’s demand for medical talents. However, most people still have certain doubts about the ability of MVE graduates. Some colleges have attempted to improve the public’s perception of MVE by modifying their school names and other means (41). In 1996, in the cultural and educational context of China’s Medical Vocational Education Law, the level of MVE began to be further clarified, and the possibility of undergraduate and graduate education was proposed. However, MVE is still mainly concentrated at the college level. MVE is often regarded as having a particular gap with traditional undergraduate medical education, particularly at the levels of social cognition and policy. It is not even considered capable of forming a competitive relationship with undergraduate education. China’s MVE has undergone more than 40 years of exploration and development. From the early days of focusing on skill training, the approach has evolved into an education model centered on personal growth and development. It aims to cultivate high-quality medical talents with innovative capabilities and support an innovation-driven society (42). Scholars have proposed a policy design for the MVE system for migrant workers. It aims to establish a sustainable MVE ecosystem by integrating the four key systems of content, certification, support, and governance (43). At the same time, some scholars have proposed an innovative development model for integrating industry, education, and cities in China from the perspective of China’s urbanization, MVE, and industrialization. This model promotes a benign interaction between urbanization, MVE, and industrialization (44). In studying the changes and reforms in medical vocational education policies, scholars analyzed the case of the Guangdong MVE Alliance. They explored the mechanism of lifelong education “credit bank” qualification recognition. The formation of this new policy network has effectively promoted the reform of medical-vocational education (45). Additionally, MVEP has evolved, exhibiting a diffusion trend in space (46). The interaction between MVE systems in different regions has a robust spatial autocorrelation (47).

Although the existing literature has provided rich theoretical support for the study of MVE, there are still some research gaps. First, there is a lack of research on the dynamic mechanism of policy changes. Existing research primarily describes the content or effects of policy in a static manner. It lacks a systematic review of the evolution of China’s MVEP, particularly an in-depth analysis of the policy stage division and the driving forces behind it. Second, the application of the theoretical framework is missing. Although some studies mention the relationship between policy and socio-economics, theoretical tools such as IAD frameworks have not been introduced, and the motivations for policy changes have not been fully explained. Third, the quantitative analysis of policy texts is weak. The existing literature primarily relies on qualitative analysis, lacks quantitative methods based on text mining, and it is challenging to objectively reveal the diachronic changes and phased characteristics of the policy center of gravity. In the field of policy research, the IAD framework has proven to be an effective theoretical tool for analyzing complex institutional and policy processes. By emphasizing self-governance, core concepts, and diagnostic methods, this framework significantly enhances the explanatory power and practical relevance of policy design, providing a structured perspective for systematic policy evaluation (48). Simultaneously, the institutional analysis perspective demonstrates advantages in comparative studies, helping to reveal how rules, practices, and values shape the structure and development paths of specific fields under different contexts (49). Employing the IAD framework allows for the systematic deconstruction of the policy “black box,” connecting exogenous variables with actor interactions. This provides solid theoretical support for in-depth analysis of the dynamic mechanisms of policy change in medical vocational education. Thus, this study focuses on the driving forces and causes of changes in China’s MVEP. Text mining and IAD theory reveal the composite dynamic mechanism of policy changes. And provide practical suggestions for the formulation of future MVEPs.

## 3 Theory and methodology

### 3.1 IAD framework

The Institutional Analysis and Development IAD framework was developed by Ostrom et al. The framework examines how natural attributes, community attributes, and institutional rules affect actors’ behavior and cumulative outcomes (24). The framework is a crucial theoretical framework in current public management and policy research, and is often employed more extensively in the study of public resources (25). It primarily focuses on the visualization and describable inference of institutional factors, thereby enhancing the research’s scientific and instructional nature (50). Its core approach is to integrate the perspectives of different fields or different disciplines to analyze how the system exerts influence on organizations and individuals, including the supply of governmental public services, the distribution of social resources, and the promotion of social equity, etc., and any field that has a system formulated by the official government to carry out the management can be analyzed using the IAD framework (51). The IAD framework has significant advantages in analyzing institutional rules and interacting with actors. However, its interpretation of informal institutions (such as cultural cognition and professional ethics) and structural power relations has limitations (24). This study employs a mixed research method to address this deficiency. The explicit change path of institutional rules is objectively presented through policy text clustering analysis and word frequency statistics.

### 3.2 Cluster analysis

This paper mainly uses text mining methods. Among various text mining methods, cluster analysis is a more advanced statistical method (52). The most commonly used methods are hierarchical and K-means cluster analysis (53). The so-called cluster analysis divides the dataset into several clusters based on the unsupervised principle, and then the subsets within the same cluster maintain a high degree of similarity (54). This study employs the K-means clustering algorithm as the foundational method for topic mining. K-means not only effectively handles large-scale textual data, but its probabilistic framework also provides a theoretical basis for identifying latent structures in complex policy discourse. Furthermore, this algorithm exhibits significant advantages in optimizing the computational process and improving analytical efficiency (55). Additionally, K-means demonstrates good adaptability and scalability in analytical scenarios that require handling multi-layered or complex interactive structures (56). Therefore, this study employs K-means clustering to systematically identify core policy themes at various stages of development (57). Specifically, the policy texts were first tokenized and stop words were removed, followed by feature extraction and vectorization using the TF-IDF method. Secondly, to overcome the influence of different feature value ranges, the TF-IDF vectors were normalized (standardized). Subsequently, the optimal number of clusters, K, was determined using the elbow method and the silhouette coefficient. Finally, to ensure the robustness of the results, the K-means algorithm was run multiple times with random initializations, and the clustering result with the best silhouette coefficient was selected for further analysis. The core formula is as follows (58).

First, choose the number of clusters K to form. K data points are randomly selected as the initial clustering center. $C_{1}$ , $C_{2}$ , …, $C_{K}$ .

Then, the clusters are assigned. For each data point $x_{i}$ , calculate its distance from each cluster center and assign it to the cluster center with the closest distance (see Equation 1).


$$\text{Assign}(x_{i})=\arg\min_{j}{\left\|x_{i}-C_{j}\right\|}^{2}$$ (1)

Finally, the centers are updated and iterated. The center of each cluster is recalculated based on the assigned clusters. Where $S_{j}$ is the set of data points in the cluster, j . Steps 3 and 4 are repeated until the center of the clusters no longer changes or changes less than a preset threshold (see Equation 2).


$$C_{j}=\frac{1}{|S_{j}|}\sum_{x_{i}\in S_{j}}x_{i}$$ (2)

In this study, a hierarchical text analysis strategy was adopted based on the scale of policy texts, the degree of thematic differentiation, and the research needs at each stage. For the initial stages, where the number of policy texts was relatively small and the themes were more concentrated, word frequency analysis was primarily used to capture the core concerns. For the quality improvement stage (2010–2020), where the number of policy texts was large, and policy discourse tended to be complex and specialized, K-means clustering analysis was applied. This allowed for more precise identification and classification of the multiple policy themes and subsystems coexisting during this stage, revealing their underlying structure. This differentiated strategy aims to match the analytical methods with the actual characteristics of policy evolution at each stage.

## 4 Results and discussion

### 4.1 The evolution of medical-vocational education policies in China

China’s medical vocational education has made remarkable progress over the past few decades. MVE’s goals, content, and training models have exhibited distinct characteristics at various stages of its history. To gain a deeper understanding of China’s MVE development process, this paper examines and analyzes China’s MVEP. The IAD framework examines the motivations and causes of policy changes, aiming to identify the shortcomings that persist in the implementation of MVEP and provide valuable insights and recommendations for formulating future policies. Furthermore, to gain a deeper understanding of the dissemination, filtering, and internalization of policy information among diverse actors, this study introduces the Bayesian Mindsponge Framework (BMF) as a complement to the IAD framework. BMF is an information processing theoretical framework focused on analyzing how individuals and organizations absorb, evaluate, and integrate new information (59). In policy process research, it helps reveal how the “information” carried by policy texts is filtered and interpreted by different actors (such as government departments, universities, and medical institutions) based on their existing cognitive frameworks (“mind sponges”), thereby influencing their strategies and interactions (60). The policy text data used in this paper comes from the “China Bailu Think Tank” website, and the content search keyword is “medical-vocational education.” The period spans from the reform and opening up in 1978 to October 2024, totaling 46 years and 10 months. In accordance with PRISMA standards (61), all policy documents (a total of 4,309) were reviewed and assessed, resulting in the elimination of irrelevant and duplicate policies. This process resulted in a selection of the most relevant policies. Ultimately, a total of 552 relevant policy documents were screened, covering all policy documents on MVE since the reform and opening up. This sample set has a reliable foundation in terms of scientific rigor, representativeness, and systematic approach. Its scientific rigor stems from its reliance on authoritative official databases. Representativeness is demonstrated by the sample’s coverage of policies at multiple levels of authority issued by legislative, administrative, and industry regulatory bodies. Systematicity is ensured through full-period retrieval and screening, using PRISMA standards, which guarantees thematic relevance and historical continuity. Of course, data collection primarily relies on publicly available written policy texts and may not fully encompass specific local, temporary, or unwritten guidelines. Furthermore, text analysis itself may not fully capture all variations and practical interactions of policies in particular implementation contexts. Nevertheless, the core set of policy texts selected for this study systematically supports the analysis of the evolution of national-level medical professional education policies.

**Figure 1:**
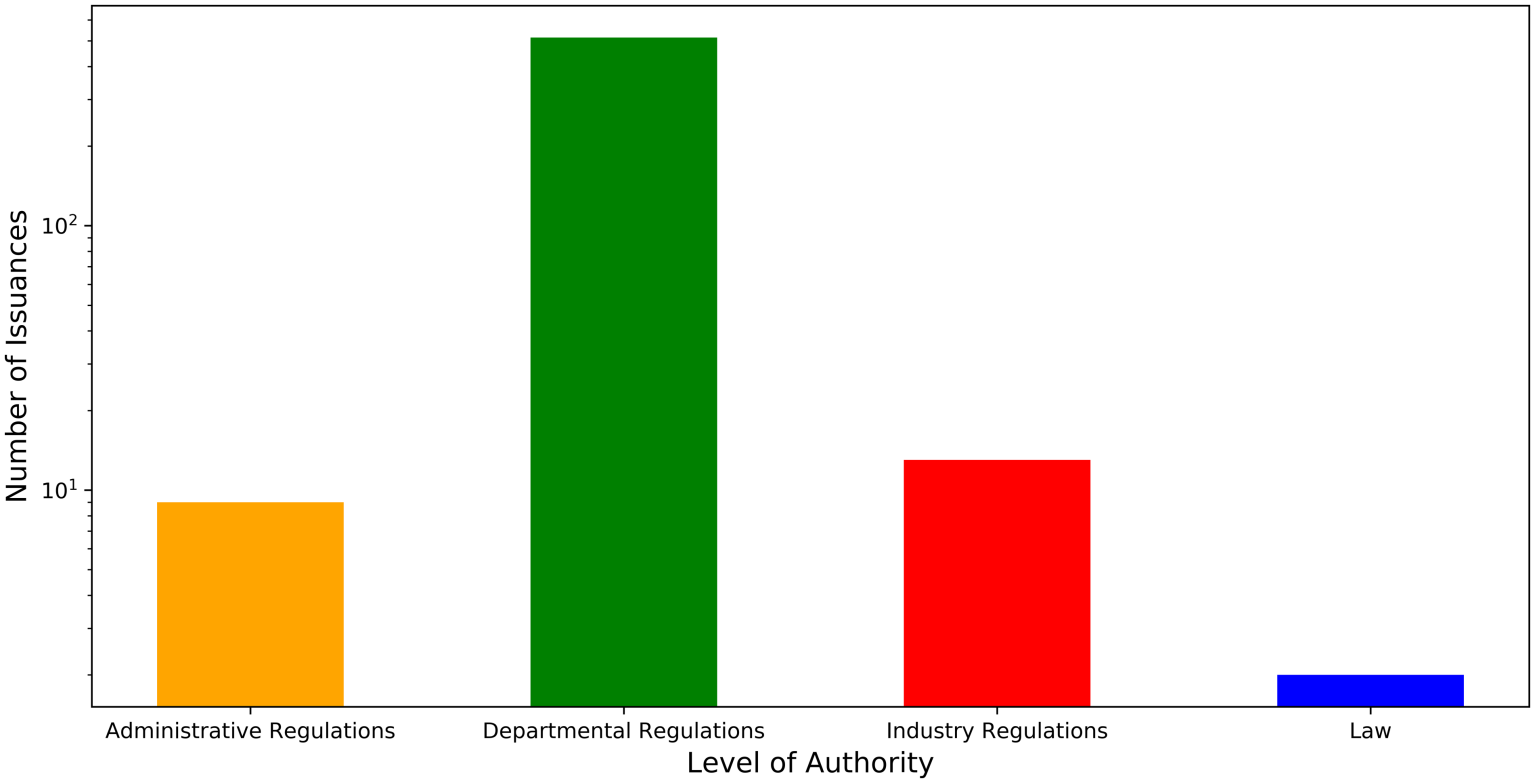
The hierarchy of policy effectiveness.

These policies can provide a more comprehensive representation of the evolution of China’s medical-vocational education policy. From the perspective of policy effectiveness, the number of policies issued by departmental regulations is the largest, followed by industry and administrative regulations. Laws account for a small proportion (Figure 1). Departmental regulations are the main form of policy change in MVE. Their characteristics are the flexibility and pertinence of policy formulation. Regulations can quickly respond to new situations and problems in MVE, making targeted adjustments and optimizations as needed. The cost of formulating departmental regulations is relatively low, and they are easy to modify and improve, which helps to form a dynamically adjusted policy system.

In addition, from the perspective of policy-making institutions (Table 1), the institutions that formulate MVEPs are characterized by diversity and hierarchy. Nationwide, policy formulation involves not only the country’s highest legislative body but also the government and its subordinate agencies, the Party Central Committee departments, and other relevant agencies. As the country’s highest legislative body, the policies on vocational education issued by the National People’s Congress have the highest legal effect. Although its issuances are relatively small, these policies often lay the legal foundation and framework for developing vocational education and provide directional guidance for formulating subsequent policies. The institutional values carried and transmitted by these policies at different levels vary significantly. These value differences, based on information entropy, profoundly influence the behavioral choices and interactions of policy participants at each level, ultimately shaping the cumulative outcomes of medical vocational education implementation (62).

**Table 1:** Distribution of policy issuances by authority.

Issuing authority	Number of issuances
National people’s congress	3
Standing committee of the national people’s congress	4
Central military commission	3
State council and its subordinate agencies	471
Central committee departments	13
Other agencies	58

**Figure 2:**
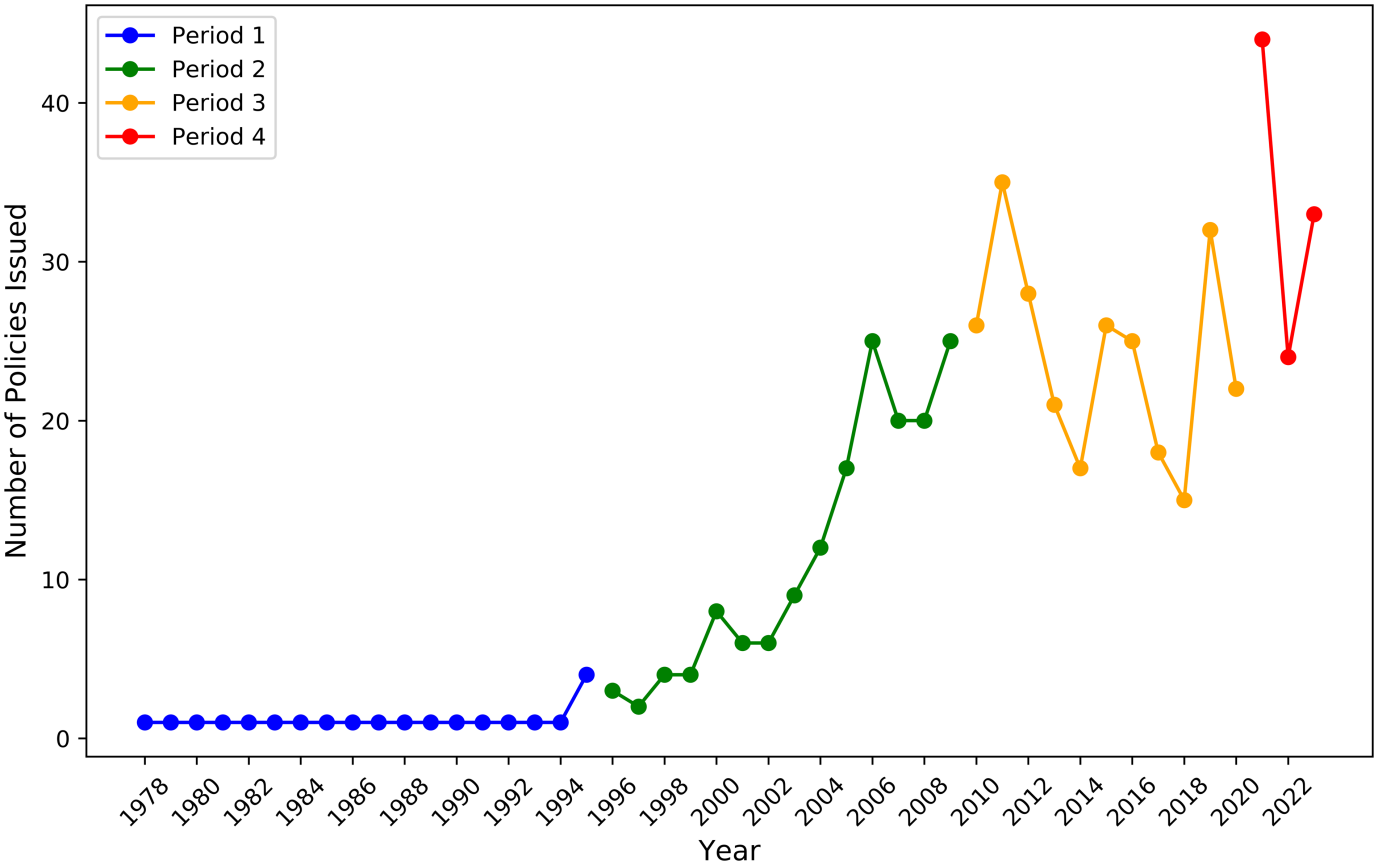
The historical evolution of medical-vocational education policies in China.

This paper categorizes China’s MVEP into stages, primarily based on the evolution and change trends of policy content, with a focus on the release of the latest policy. Policies typically exhibit a certain degree of continuity, but new policies following reform often differ significantly from their predecessors (63). As an essential part of the national education system, MVE must be adjusted accordingly as national development strategies and social and economic needs change. In different historical periods, the formulation and implementation of MVEPs aim to cultivate skilled medical professionals who can adapt to the evolving needs of the times and promote the advancement of society and the medical industry. Therefore, the stage division of MVEPs reflects the evolution of education policies and the changes in national development strategies and social needs (64). To better understand the focus of policy at different stages, high-frequency words in policy texts are used to identify the core content of the policies. Words that appear frequently in policy documents indicate that the policy attaches great importance to its field. Through an in-depth analysis of 552 MVEP documents, combined with the socioeconomic context of different periods, China’s MVEP is divided into four periods (Figure 2). The core basis for dividing the stages is the significant shift in policy paradigms. (1) The promulgation of landmark laws or national strategic documents (such as the 1996 Vocational Education Law, the 2010 National Medium- and Long-Term Education Reform and Development Plan, and the 2021 “Quality Improvement and Talent Cultivation Action Plan”) constitutes constitutional turning points in institutional change. (2) The clustered changes in high-frequency keywords in policy texts reflect the shift in focus of core policy issues and tools. (3) The phased characteristics of the number and intensity of policy releases reflect the rhythmic changes in national resource investment and attention. Based on the above criteria, the breakpoints of the four stages can be clearly identified. From an overall development perspective, the number of policies promulgated in the first period was relatively small. In contrast, the number of policy releases gradually increased since the second period and peaked in the fourth. It can be foreseen that the number of MVEPs will continue to grow to meet the new challenges and opportunities facing medical education (65).

#### 4.1.1 Preliminary development stage (1978–1995)

After the reform and opening up, economic construction became China’s central task. Economic development requires a large number of skilled workers and professionals, particularly those in the medical field. Faced with growing medical needs and public health challenges, medical vocational education has become a key factor in cultivating medical and healthcare talents. However, China’s education system was mainly single at that time, especially since the development of MVE was relatively backward and could not meet society’s and the economy’s needs. Although many medical graduates possess basic theoretical knowledge, due to a lack of systematic professional practice experience, many hospitals and medical institutions struggle to recruit qualified professional and technical personnel. Overall, there appears to be a significant supply shortage in the medical labor market. Therefore, the state has increased its support for MVE, adjusted the structure of medical education, and promoted the rapid development of medical technology education. In 1978, the state issued a series of policies, including the “Policy on Adjusting the Structure of Secondary Education” and the “Decision of the CPC Central Committee on Reforming the Education System,” which outlined the direction for developing MVE. Under the guidance of these policies, the proportion of medical professionals and technical personnel receiving training has been gradually increased, and the level and model of medical education have been adjusted accordingly. In particular, in terms of secondary medical education, ordinary middle schools have been gradually transformed into medical vocational schools, health schools, nursing schools, and other similar institutions to strengthen the foundation of medical technical education. At the same time, the state advocates for the coexistence of full-time, part-time work-study, and non-formal education. This reform has laid the foundation for the diversified development of MVE. Additionally, the policy is proposed to strengthen financial support for MVE and enhance school conditions. It also focused on improving the teaching staff of medical education and promoting in-depth cooperation between medical schools and medical institutions. It strives to cultivate more high-quality medical talents with clinical experience and operational skills (66).

**Figure 3:**
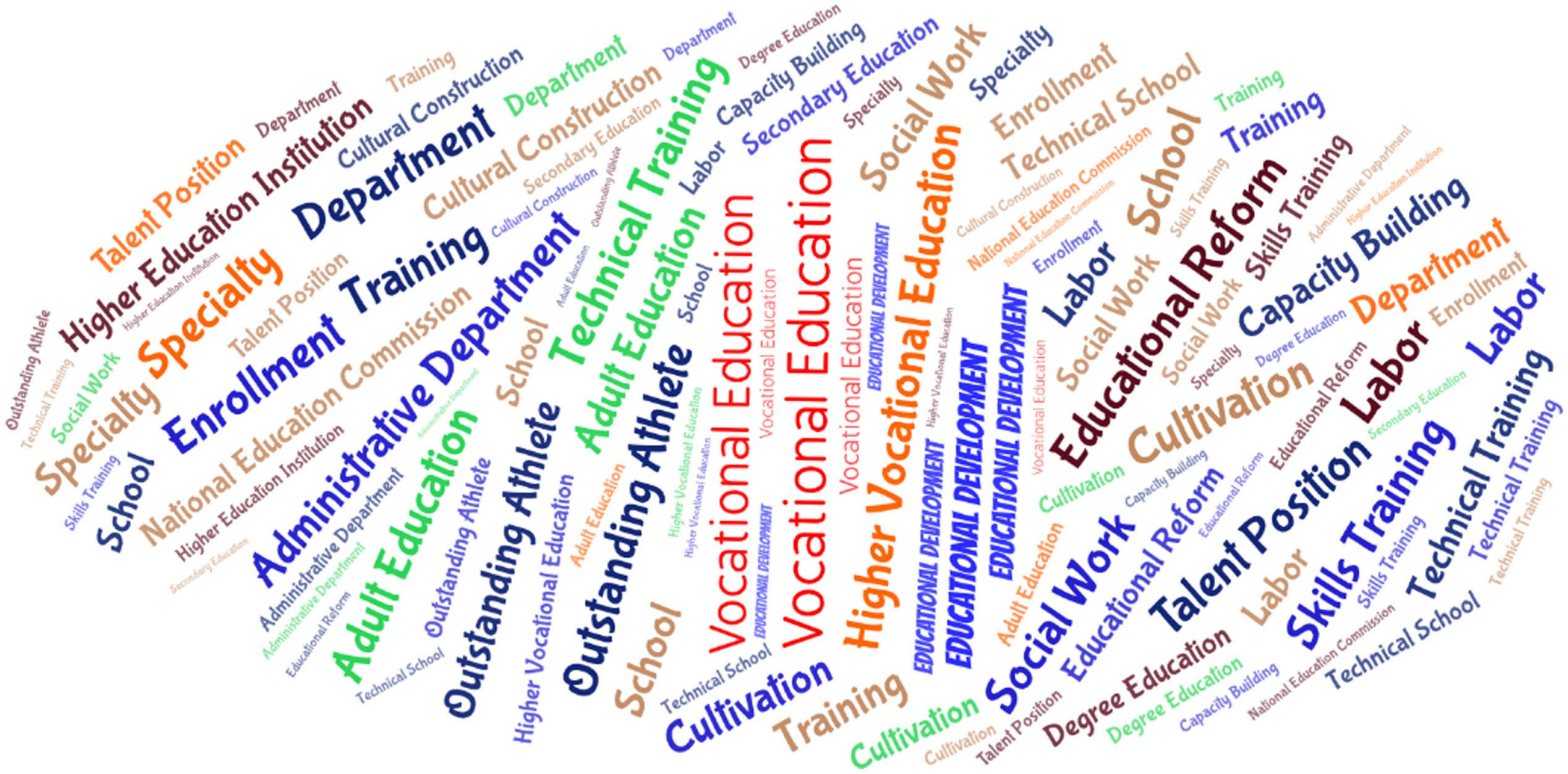
Policy word frequency chart in the preliminary development stage.

As can be seen from the high-frequency words in the policy texts of this stage (Figure 3), “Vocational Education,” “Educational Development,” and “Higher Vocational Education” indicate that the policy of this stage aims to improve the overall level of medical-vocational education, especially in higher medical vocational education. “Skills Training,” “Technical Training,” and “Training” indicate that the policy focuses on enhancing the skills and technical proficiency of the medical workforce to meet the increasing demands in the medical and healthcare field. “Secondary Education” and “Adult Education” demonstrate that the policy prioritizes establishing a multi-level MVE system that addresses the educational needs of various age groups and levels. Some regions have begun implementing the “training first, employment later” orientation. Reform the medical graduate allocation system, encourage medical vocational school graduates to choose their jobs, and promote the implementation of the medical professional qualification certificate system.

However, MVE is still in its early stages of development. Some schools have poor operating conditions and weak teaching staff, making it challenging to ensure the quality of education. The reasons are as follows: first, although the state has increased its funding for MVE, it is still insufficient overall. Some medical schools’ teaching facilities and internship conditions are poor, making it challenging to implement practical teaching. Second, the management system and operating mechanism of MVE are still imperfect, and the development model of industry-education integration and medical-school cooperation needs to be further deepened and improved. In particular, a significant gap remains in the collaboration between medical institutions and medical schools. Third, MVE has a low degree of recognition in society, and some students and parents still prefer to choose general higher education and other traditional medical schools. MVE is not sufficiently attractive, especially in certain regions where the social recognition of medical vocational education is relatively low (43).

#### 4.1.2 Legalization stage (1996–2009)

The period from 1996 to 2009 was crucial for the legalization of medical vocational education in China. During this period, China’s economy continued to develop rapidly, and the demand for high-quality medical and technical personnel in response to people’s health needs and advancements in medical technology increased dramatically. The country attaches great importance to the development of MVE. It promotes quality improvement and enhances the MVE system through a series of policies and legal documents. In 1996, the promulgation of the Vocational Education Law of the People’s Republic of China marked the beginning of a new era of legal governance of MVE in China. The law clarifies the legal status of MVE and stipulates the responsibilities of the government and relevant departments in relation to MVE. In February 1998, the State Education Commission issued the Opinions on Accelerating the Reform and Development of Vocational Education in Central and Western Regions, with a particular emphasis on supporting MVE in remote Western regions. It aims to narrow the gap in the development of MVE in different areas and improve medical education in Western regions. In July 2001, the General Office of the Ministry of Education promulgated the Modern Distance Medical Vocational Education and Adult Education Resource Construction Project. It actively promotes the integration of modern distance education and MVE to provide more opportunities for medical education in remote areas. In 2003, the Ministry of Education established the project team for “Feasibility Analysis and Research on the Reform of the Curriculum System of Higher Medical Vocational Education,” which opened a new chapter in developing higher MVE in China. During this period, MVE gradually established a multi-level and multi-field system for training medical technical personnel from primary medicine to advanced medical technical personnel. Higher MVE combines general medical education and medical research, especially in cultivating clinical skills, medical ethics, and communication skills, forming a closer coordination mechanism. The policy emphasizes the close integration of MVE with social and economic development, promotes the integration of industry and education, and promotes cooperation between medical schools. It also cultivates high-quality medical and technical personnel who can adapt to the needs of the medical industry. The government encourages industries, medical institutions, social groups, and individual citizens to participate in the organization and management of MVE by law, forming a diversified school-running pattern. The policy at this stage also pays increasing attention to the employment orientation of MVE, emphasizing the adaptability and employment ability of medical professionals, and promotes the development of a medical-related professional qualification certification system (42).

**Figure 4:**
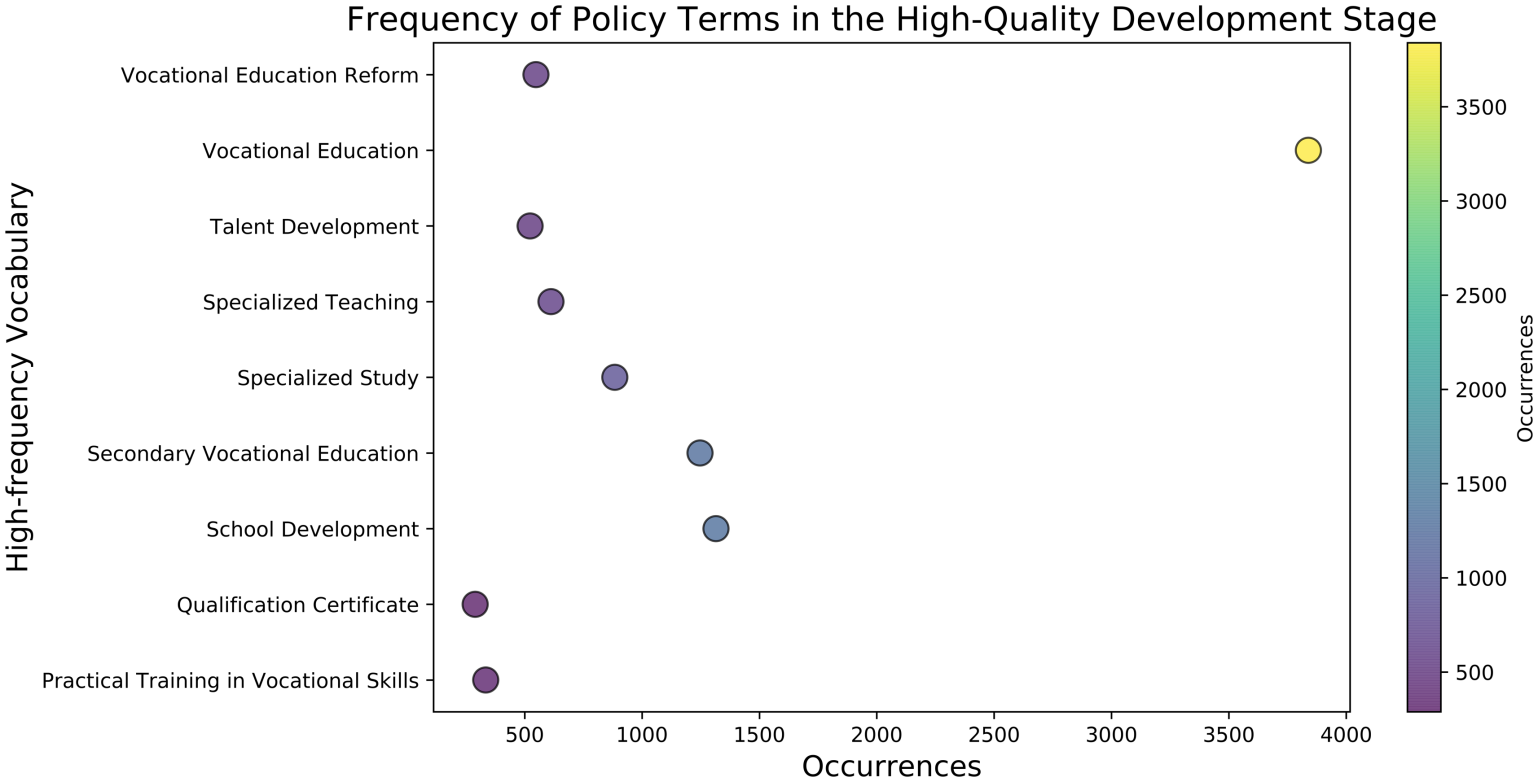
Frequency of policy terms in the legalization stage.

Judging from the high-frequency words in the policy texts of this stage (Figure 4), “School Development” indicates that the conditions of MVE institutions are constantly improving. This includes improving infrastructure, updating the curriculum, and rationally allocating resources to promote a better learning environment, particularly for the unique needs of the medical profession. The “Vocational Education Reform” reflects that MVE is undergoing a process of change and adjustment, primarily to meet the evolving needs of the medical industry and the changing health needs of society. “Specialized Study” and “Specialized Teaching” represent a customized educational approach for specific medical skills. This specialization is essential to ensure that medical students acquire in-depth knowledge and practical skills that are consistent with the requirements of the medical industry. “Qualification Certificate” shows the importance of certification in MVE. These certificates, as verification of medical skills and knowledge, not only enhance the employability of graduates but also provide more opportunities for their career development. Nevertheless, there are still some shortcomings in developing MVE at this stage, especially regarding resource allocation. There is an imbalance in MVE resources between different regions and different types of medical education institutions, especially in rural and remote areas, where medical vocational education resources are relatively scarce. The quality gap in medical education is pronounced in these areas, affecting the overall quality improvement of doctors and medical technicians. In addition, implementing medical school cooperation in MVE still faces numerous difficulties and challenges, particularly in clinical internships, scientific research collaborations, and other areas of cooperation. The enthusiasm of enterprises and medical institutions to participate in MVE is relatively low, which limits the expansion and improvement of medical education practice (67).

#### 4.1.3 Quality enhancement stage (2010–2020)

Since 2010, China has been in a critical period of economic restructuring and industrial upgrading. With the rapid development of modern medical and healthcare services, particularly the continuous advancement of medical technology and public health services, the demand for high-quality medical professionals is increasing. At the same time, the accelerated urbanization process and the unbalanced layout of urban and rural medical resources have led to an urgent need for high-skilled medical talents in the medical industry. The country attaches great importance to the development of MVE. It regards it as an essential means to improve the quality of medical services, protect public health, and promote economic growth. Therefore, the government has issued policy documents covering all aspects of MVE, including training medical students, clinical practice, internship guarantees, and curriculum reform, demonstrating the importance of MVE. In 2010, the release of the Outline of the National Medium- and Long-Term Education Reform and Development Plan (2010–2020) proposed building a modern vocational education system and including MVE in it. The outline emphasizes the importance of improving the quality of medical education. It proposes to enhance the training of medical professionals, particularly in terms of medical skills and clinical practice capabilities. In 2014, the “Decision of the State Council on Accelerating the Development of Modern Vocational Education” clearly pointed out that it is necessary to accelerate the improvement of the MVE system. Specific measures, such as strengthening medical practice teaching and optimizing the allocation of medical education resources, were proposed. This policy responded to the growing demand for medical talents and laid the foundation for the reform of MVE. In 2017, the State Council issued the “Several Opinions of the General Office of the State Council on Deepening the Integration of Industry and Education,” which clarified the deep integration of MVE and the medical industry. The policy advocates for cooperation between medical schools and medical institutions, promotes the integration of industry, academia, and research, strengthens clinical practice and medical technology training, and enhances the practical operational ability of medical students. Especially in clinical medicine, nursing, and public health, the policy proposes strengthening school-hospital cooperation and interdisciplinary integration to enhance the comprehensive quality of medical education for students. In 2019, the Ministry of Education issued a notice on the in-depth study and implementation of the “National Vocational Education Reform Implementation Plan,” marking the beginning of a more in-depth implementation stage for the reform of MVE. The notice clarified the reform direction of MVE and proposed a close connection between MVE and social needs. It emphasized cultivating medical talents with an innovative spirit and practical ability. In particular, in promoting cooperation between medical schools, hospitals, and pharmaceutical companies, the policy further strengthened the collaboration between medical schools and proposed innovative talent training models. This model enhances medical students’ clinical practice skills and ability to solve practical problems, meeting the development needs of the modern medical and healthcare industry (19).

**Figure 5:**
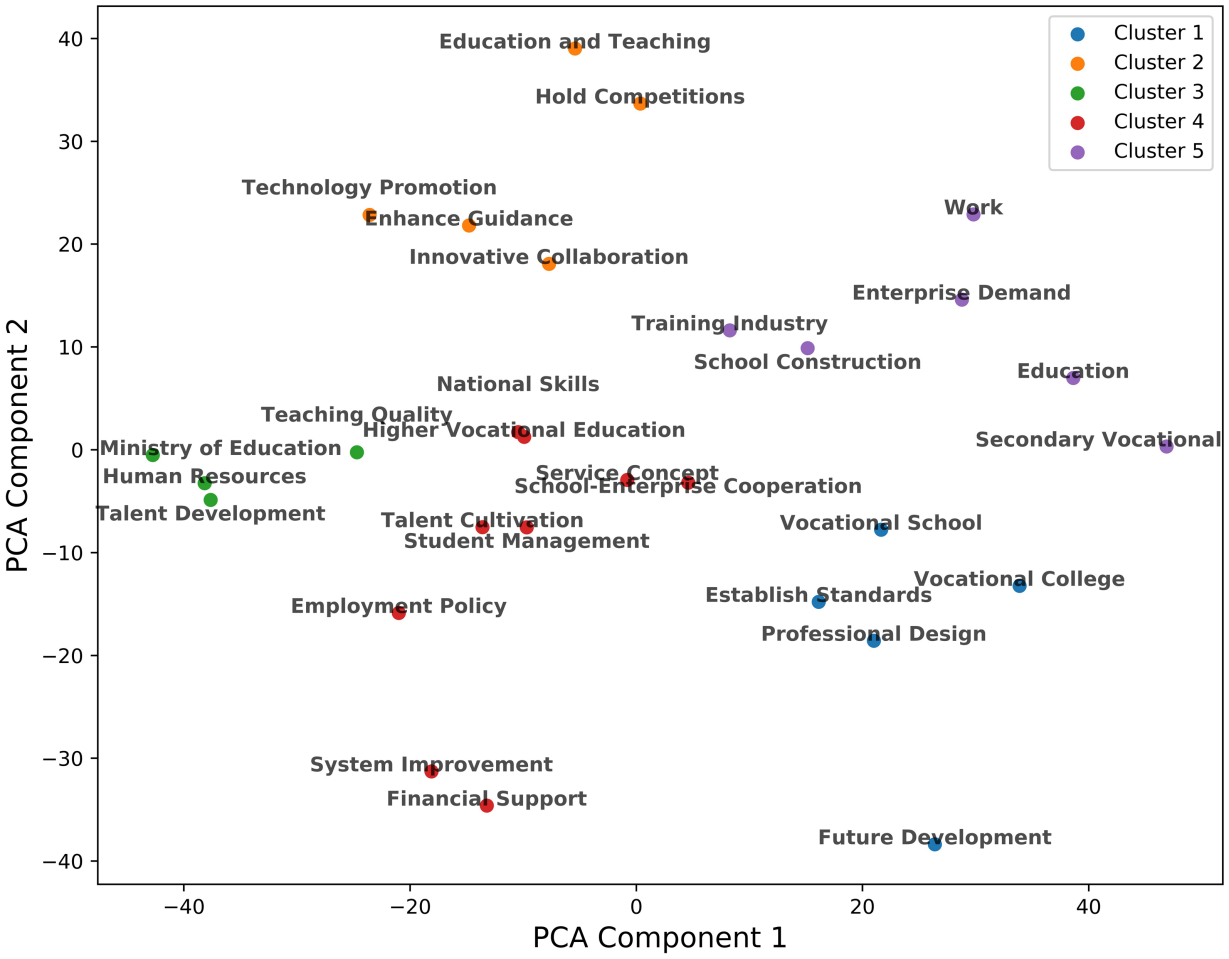
Keyword clustering of policies in the quality improvement stage.

This stage witnessed a peak in the number of policy releases, with policies broadly covering multiple dimensions, including professional development, teaching reform, school-enterprise cooperation, and talent cultivation. The policy discourse system became increasingly complex. To go beyond simple word frequency analysis and delve into the complex structure of coexisting and intertwined policy themes during this period, this study employs the K-means clustering analysis method in this section explicitly. This method can objectively identify intrinsic thematic clusters from a large amount of text data. This helps to systematically reveal the multi-dimensional policy framework constructed by the state in promoting the “quality improvement” of medical vocational education. From the cluster analysis of the medical vocational education policy texts in this phase (Figure 5), the relevant phrases can be divided into several key categories. “Vocational School,” “Vocational College,” “Establish Standards,” and “Professional Design” form Cluster 1, which is labeled as “Professional Construction.” This shows the close connection between MVE and skills training. The policy focuses on cultivating medical talents with professional skills through medical schools and professional settings, and enhancing students’ professional capabilities to meet the growing demand for talent in the medical industry. “Education and Teaching”, “Hold Competitions”, “Technology Promotion”, “Enhance Guidance”, “Innovative Collaboration”, and others constitute Cluster 2, marked as “Teaching Reform.” This category reflects the measures taken by policies to promote MVE reform and development. Reform and innovation are recognized as the primary driving forces for enhancing the quality of education. This aims to strengthen the promotion of science and technology in education and teaching. “Talent Development,” “Human Resources,” “Ministry of Education”, and so on constitute Cluster 3, marked as “Talent Resources.” This category is based on the country’s consideration of its human resources. Medical vocational education is a significant strategy for national development. Therefore, the Ministry of Education will invest heavily in this field. “School-Enterprise Cooperation”, “Service Concept”, “Employment Policy”, “Financial Support”, and so on constitute Cluster 4, marked as “School-Enterprise Cooperation.” This category shows the close cooperative relationship between medical schools and medical institutions. Medical and school cooperation aims to better align with the needs of the medical industry through the implementation of actual projects. Medical students are exposed to a real medical environment and industry needs during their learning process, which can enhance their competitiveness in the job market. “Training Industry”, “Enterprise Demand”, “Work”, and so on constitute Cluster 5 and are marked as “Market Demand.” This category focuses on the market’s demand for medical professionals. The birth of many medical training industries can help medical students become more competent. To assess the internal consistency and construct validity of the clustering results, the silhouette coefficient was calculated for each cluster. In this analysis, the mean silhouette coefficient for all clusters was 0.67 (range: 0.62–0.74). This result indicates that phrases within clusters have high semantic consistency, and the separation between categories is good, suggesting that the clustering scheme is statistically robust.

Although MVE has achieved remarkable results at this stage, it still faces some shortcomings. First, the training conditions need to be improved. Although the central government has supported the construction of medical training bases, some schools’ training equipment and conditions remain imperfect, which affects practical teaching. Second, the connection between student employment and the program is not sufficient. Although the policy emphasizes cooperation between medical schools, in actual operation, there are still problems with the connection between students’ internships and employment, and the medical school cooperation mechanism needs further improvement (68).

#### 4.1.4 High-quality development stage (2021 to present)

Since 2021, China’s medical-vocational education has entered a stage of high-quality development. Due to the increasing demand for medical services, social problems such as an aging population, and the rapid advancement of science and technology, China’s medical industry faces new development opportunities and challenges. The salient feature of this stage is the transformation and upgrading of the medical industry, especially in technological innovation, precision medicine, and health management, which urgently need a large number of high-quality medical professionals. However, the quantity and quality of talents cultivated by traditional MVE are insufficient to meet this demand. Therefore, the country has gradually increased its attention to MVE. MVE is crucial to the national education system and the development of medical human resources. It shoulders the important task of cultivating multi-level and multi-field medical professionals, inheriting medical and technical skills, promoting medical employment, and promoting medical reform. In the new journey of building a modern socialist country in an all-around way, MVE has broad prospects and essential development potential. In 2021, the Ministry of Education issued the “Action Plan for Improving the Quality and Cultivating Excellence in Medical Vocational Education (2020–2023)”, announcing that MVE has entered a stage of high-quality development. The “Fund Management Measures for the Quality Improvement Plan for Modern Medical Vocational Education” was promulgated in the same year. This provided financial support for the quality improvement of MVE. In 2023, the Ministry of Education issued the “Implementation Plan on Further Deepening the Integration of Industry and Education in Medical Vocational Education to Serve the High-quality Development of the National Health Career.” The plan aims to foster an organic connection between medical education and the medical industry, as well as scientific and technological innovation, by integrating industry and education. On the other hand, it aims to promote transformation and upgrading in the medical field, achieving high-quality development of the medical and healthcare industry. Deepen the integration of industry and education, as well as cooperation between medical schools in MVE, to foster a pattern of mutually beneficial interaction between medical education and industrial demand, thereby enhancing the quality of medical talent training and social adaptability. At this stage, the MVE system is more comprehensive, encompassing multiple levels, including secondary vocational education, higher vocational education, and undergraduate education. At the same time, the internationalization of medical education has accelerated. Through Sino-foreign cooperative education and international exchanges, the global influence of China’s MVE has been enhanced, and more international perspectives and advanced educational concepts have been injected into the country’s MVE (69).

**Figure 6:**
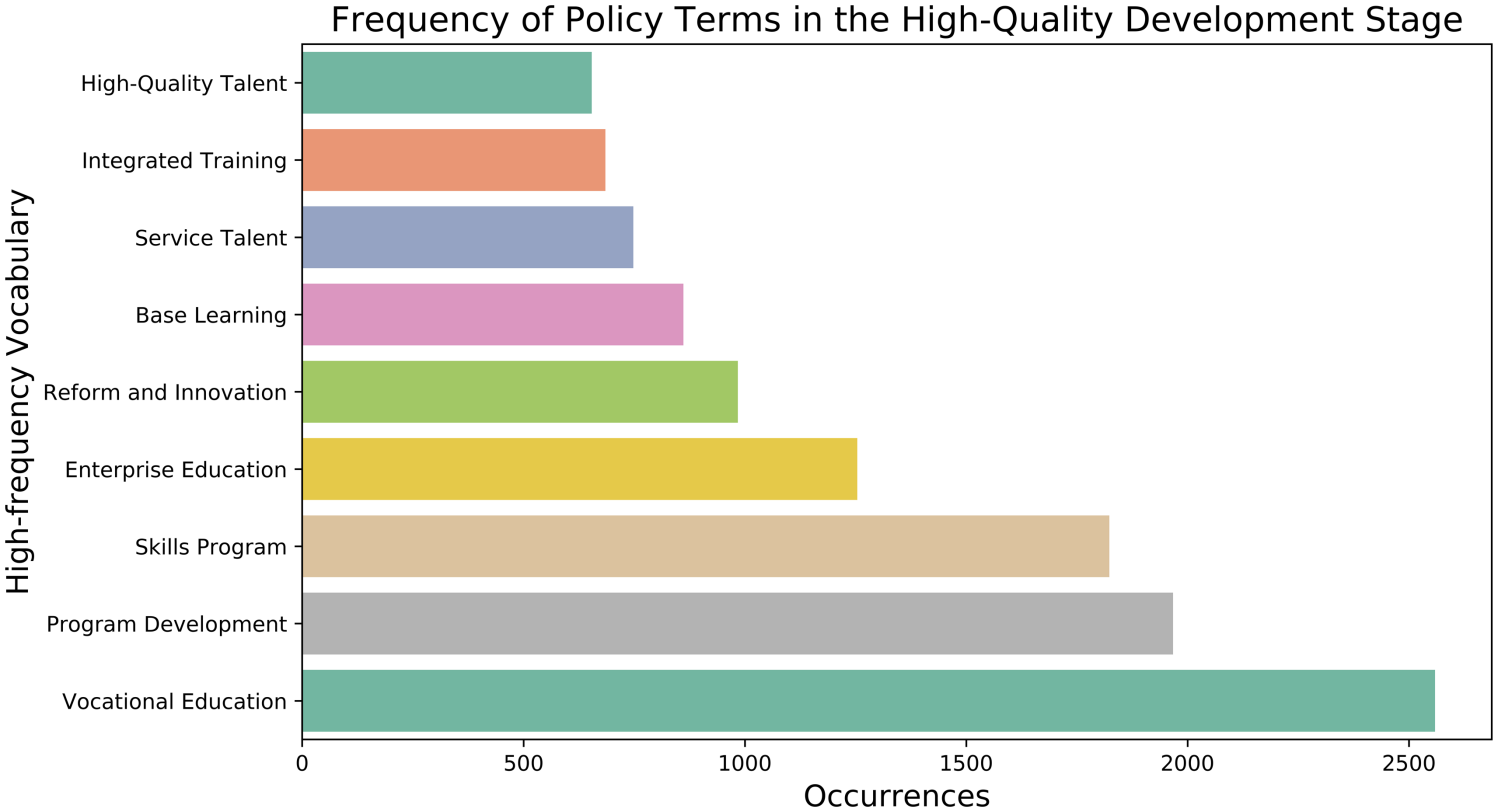
Frequency of policy terms in the high-quality development stage.

Judging from the high-frequency words in the policy texts of this stage (Figure 6), “Program Development” refers to establishing and optimizing various educational programs in MVE to ensure that these programs meet the standards of the medical industry and address market needs. This includes setting medical courses and improving teaching methods and evaluation systems. For example, the curriculum design of medical higher vocational and secondary vocational education must be aligned with the requirements of clinical practice and essential medical knowledge. This ensures that the trained medical talents can adapt to the specific needs of different medical environments. “Reform and Innovation” reflects the policy’s aim to enhance the adaptability and foresight of MVE through institutional reform and educational innovation. Specifically, adjusting policies and regulations, reforming the school-running system, and updating academic content are all aimed at enhancing the flexibility and modernization of medical education to adapt to the rapid advancements in medical technology and evolving clinical needs. “Integrated Training” emphasizes the diversity and flexibility of MVE, combining theoretical learning with practical skills through an integrated training model, particularly in designing clinical skills training and internship links, to enhance students’ comprehensive quality and employability. The “dual certificate system” of medical education (skill certificate and academic certificate) also reflects this training model, which aims to cultivate students’ clinical practice ability and medical skills to better work in hospitals, clinics, and other medical institutions. The goal of “High-Quality Talent” is to cultivate high-quality medical professionals with innovative abilities, practical skills, and professional ethics to meet the needs of economic development and social progress. Medical professionals require a solid foundation in professional knowledge, technical expertise, sound professional ethics, a teamwork spirit, and an exceptionally comprehensive ability in patient management, doctor-patient communication, and other relevant aspects. Overall, the quality of talent training in China’s MVE has improved significantly. However, there are still some challenges in the actual implementation. In particular, the MVE resources in the central and western regions are relatively scarce, resulting in limited medical talent training capacity. Additionally, the construction of “dual-qualified” teaching staff in MVE still requires strengthening, and teachers’ clinical experience and practical abilities must be enhanced. Medical graduates’ employment channels and career development opportunities must also be broadened, especially in primary medical institutions and rural medical systems. To this end, policies should strengthen the allocation of resources for medical education in different regions and levels to promote the balanced development of medical education (70).

### 4.2 Analysis of the evolution of medical-vocational education policies

**Figure 7:**
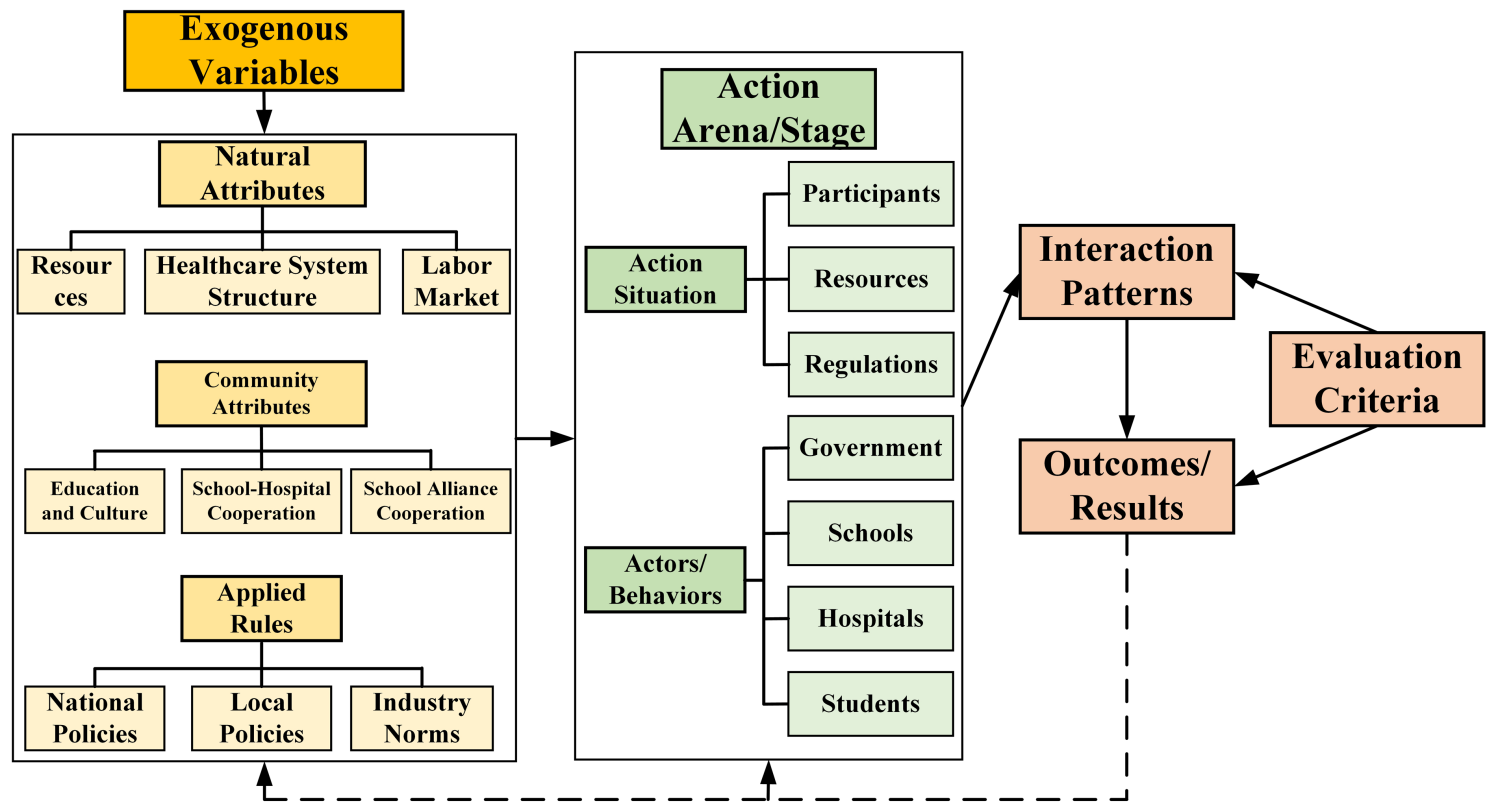
The IAD framework for analyzing medical-vocational education policy change.

The Institutional Analysis and Development (IAD) framework was developed by Ostrom et al. (26). This framework examines how natural attributes, community attributes, and institutional rules influence the behavior of actors and their cumulative outcomes. This essential theoretical framework, widely used in current public management and policy research, is employed in the study of public resources (25). It mainly makes institutional factors visual and describable, which improves the scientific nature and guidance of the research (50). Its core method is to comprehensively analyze how institutions influence organizations and individuals from the perspectives of different fields or disciplines. This includes issues such as the supply of government public services, allocating social resources, and promoting social equity. Any field managed by an official government-established system can be analyzed using the IAD framework. Based on the original IAD framework, this paper constructs an IAD framework suitable for studying the policy changes in MVE in China. It provides a theoretical basis for in-depth research on the causes and paths of policy changes in MVE (Figure 7). Through this framework, we can identify and analyze the critical factors of policy changes in MVE. We can examine how these factors interact in various historical and social contexts to influence the design, implementation, and effectiveness of medical education institutions.

#### 4.2.1 From preliminary development to legalization stage

##### 4.2.1.1 Analysis of natural attributes and community attributes

In the initial development stage, China’s MVEP was mainly driven by the urgent need for economic recovery and growth in the early stage of reform and opening up. With the transformation of the economy and the acceleration of urbanization, the MVE system has been required to reform. From the perspective of exogenous variables and natural attributes, during this period, the introduction of the market economy led MVE to cease serving the traditional medical industry and instead adapt to the needs of emerging medical fields and modern medical services. For example, with the rapid development of information technology, the establishment of related majors such as medical informatics and digital medicine has gradually increased. The urbanization process has promoted changes in the labor market. Many rural laborers have migrated to cities, resulting in the concentration of demand for medical and healthcare services in urban areas. MVE must quickly adjust to cope with this change. From the perspective of community attributes, society’s understanding of MVE has gradually deepened, which has improved the social status of MVE and laid the groundwork for the legalization stage (24). Therefore, from the perspective of the IAD framework, the initial driving force behind this transitional phase stems from changes in natural material conditions (economic transformation and urbanization), which in turn reshaped the community’s needs and perceptions regarding medical professional education. The key issue in the action arena shifted from meeting the quantitative demand for basic skilled personnel to establishing a stable and reliable system for supplying medical skills.

##### 4.2.1.2 Analysis of application rules

From the perspective of application rules, the Vocational Education Law of the People’s Republic of China was promulgated in 1996. This education law protects MVE and clarifies the responsibilities and obligations of the government, schools, and medical institutions. Establishing this legal framework marks the transformation of China’s MVE from a “policy-oriented” to a “rule of law-oriented” approach, laying the foundation for subsequent reform of the MVE. However, despite the initial establishment of the legal framework, MVE still faces numerous challenges, including low social recognition and difficulties in securing employment for students. Against this background, the government, schools, and medical institutions have begun to explore new cooperation models under the legal framework. The cooperation mechanism is not yet mature and needs further improvement. From the perspective of multi-level systems, the transformation of this stage reflects significant institutional construction characteristics (71). At the operational level, policies began to focus on specific measures such as internship guarantees, teaching reform, and the construction of training bases for MVE. These measures aim to enhance the practicality and applicability of MVE, laying a solid foundation for students’ future medical careers. At the collective level, policymakers began exploring new models for the deep integration of MVE and the medical industry. For example, school-medicine cooperation and work-study alternation are introduced to promote two-way interaction between education and the medical sector, improving the pertinence and effectiveness of MVE. At the constitutional level, the government has strengthened macro-guidance and supervision of MVE by establishing a multi-level MVE system that covers the educational needs of different age groups and levels (25). This series of rule adjustments indicates that policymakers are attempting to regulate and guide the specific activities at the operational level (teaching and internships) through changes at the constitutional choice level (legislation) and the collective choice level (exploration of cooperative models), in response to pressures arising from natural and social attributes.

##### 4.2.1.3 Comprehensive analysis

This stage reflects the mandatory change due to the imbalance between institutional supply and demand. To begin with, the coupling between institutional gaps and opportunity windows. The differentiation of medical service formats brought about by the market economy has prompted MVE to move toward institutionalized construction. The promulgation of the Vocational Education Law reconstructs the rules system of MVE, essentially an administrative absorption of spontaneous market demand. Then, the asymmetric evolution of multi-level institutions. The constitutional selection layer is ahead of schedule, making it “hard to follow the law.” The collective selection layer is path-dependent, and local governments are more willing to invest in higher education than vocational education due to GDP assessment orientation. Lastly, the game of actors and the power of power are reconstructed. The essential essence of the legalization process is the redistribution of power by stakeholders (government, colleges, and medical institutions). Although the country advocates improving the status of MVE, society is stubbornly aware that “vocational education is inferior to others.” This reflects the locking effect of community cognition. In summary, this transformation is essentially a process of mandatory institutional construction within the IAD framework, driven by exogenous variables (market economy), where the rule system in the action arena shifts from “absence” to “establishment.” However, the tension between the newly established formal rules (applied rules) and deeper community attributes (social norms) leads to challenges in implementation, revealing the complexity and path-dependent characteristics of institutional change.

#### 4.2.2 From legalization to quality improvement stage

##### 4.2.2.1 Analysis of natural attributes and community attributes

According to the IAD framework, once the fundamental application rules (laws) are established, the focus of policy change shifts to how to make the rule system more effectively adapt to new exogenous variables. The legalization stage provides a legal basis for MVE. With the continuous growth of the economy and the optimization and upgrading of the industrial structure, MVE faces new challenges and opportunities. From the perspective of exogenous variables and natural attributes, technological progress and changes in industrial layout have presented new requirements for the content and teaching methods of MVE (25). The continuous growth of the economy and the optimization and upgrading of the industrial structure require MVE to cultivate highly skilled medical talents with innovative ability, practical ability, and international vision. With changes in population structure and intensified competition in the labor market, improving the quality of medical workers has become a key factor in enhancing national competitiveness. From the perspective of community attributes, the increasing demand for cooperation between the medical industry and educational institutions has promoted the deep integration of MVE and the medical sector, providing strong support for improving the quality of education (72).

##### 4.2.2.2 Analysis of application rules

From the perspective of application rules, MVE still faces problems such as uneven distribution of educational resources and uneven educational quality (50). Therefore, policymakers have begun to focus on specific measures, such as internship guarantees, educational reform, and the establishment of training facilities, to enhance the quality of education. From the multi-level systems perspective, the government, medical schools, and medical institutions seek deeper cooperation under the quality improvement framework to promote the high-quality development of MVE jointly. At the operational selection level, policies focus on improving curriculum setting, teaching methods, and training bases, as well as enhancing the teaching quality and effectiveness of MVE by introducing advanced teaching concepts and technical means. At the collective selection level, policymakers continue to develop new models of integrating MVE and the medical industry, such as industry-education integration and medical school cooperation. The deepening of these models aims to promote the integration of education and the medical sector, improving the social recognition and influence of MVE. The government continues to strengthen macro-guidance and supervise MVE at the constitutional level. It provides a more powerful institutional guarantee for developing MVE by improving relevant laws, regulations, and policy documents (25). The refinement of the rule system at this stage reflects the deep interaction between operational choices and collective choices within the IAD framework. Policies implement quality requirements through specific measures (at the operational level), and the effectiveness of these measures depends on the deepening of collective action models, such as collaboration between medical institutions and universities.

##### 4.2.2.3 Comprehensive analysis

This stage essentially reflects the adaptive changes of the institutional system to changes in the external environment. To begin with, there is a conflict between institutional rigidity and environmental elasticity. The rapid innovation of medical technology has led to a disconnection between existing educational standards and the needs of clinical practice. The policy has been designed through flexible rules, such as “dual-teacher” teacher certification and modular course development, to increase institutional flexibility within the legal framework. Next, the central government strengthened its vertical control over MVE through special fiscal measures. Still, local governments tended toward ordinary colleges and universities due to financial pressure, which created a power struggle for allocating resources between central and local areas. Lastly, the contract for school-enterprise cooperation has encountered difficulties, and the integration of industry and education has become a formality. Thus, this stage of transformation reflects the adaptive adjustment process within the IAD framework. The rule system (applied rules), under the existing legal framework (constitutional choices), enhances its resilience by introducing flexible mechanisms to cope with challenges arising from natural attributes such as technological change. However, the resource competition and contractual dilemmas among actors highlight the complexity of interest coordination within the community, which is a key factor affecting institutional performance.

#### 4.2.3 From quality improvement to high-quality development stage

##### 4.2.3.1 Analysis of natural attributes and community attributes

At the beginning of the 21st century, as China’s economic growth model shifted from extensive to intensive, the social and financial requirements for the quality of technical talent continued to increase. This also prompted policies to pay more attention to the development of MVE. In 2022, the newly revised “Vocational Education Law” clarified the positioning of vocational education as a type of education. It opened up the connection between secondary vocational education, higher vocational education, undergraduate education, and graduate education, thereby improving the modern vocational education system. It provides students with multiple choices, convenient and flexible learning conditions, and opportunities, enhancing the “broken head” phenomenon in MVE. From the perspective of external variables and natural attributes, the weakening of the demographic dividend, the decline in birth rate, and the intensification of the aging problem have made improving the quality of practitioners in the medical field the key to sustainable economic development. Society’s expectations for high-quality MVE are gradually increasing, and the public’s awareness of MVE is constantly improving. Increasingly, people are paying attention to medical technology and skills training, and MVE is gradually being regarded as an essential pillar of social and economic development. From the perspective of community attributes and interaction patterns, the government, schools, and medical institutions are seeking deeper cooperation within the framework of high-quality development. Frequent interactions and the formation of a suitable cooperation mechanism jointly promote the high-quality development of MVE. The country actively promotes “industry-education integration” and “medical-school cooperation.” It encourages medical institutions to participate in designing and implementing MVE courses, ensuring that the educational content is aligned with industry needs and requirements. In addition, medical vocational schools are also constantly exploring innovative teaching models, such as project-based learning and case-based teaching, to enhance students’ clinical practice abilities and comprehensive quality (73). At this stage, exogenous variables in the IAD framework (such as national strategies and international standards) exert a more substantial shaping influence, driving the evolution of community attributes toward “networked collaboration.” The interaction patterns within the action arena shift from a linear “government-school-enterprise” relationship to a multi-node, deeply coupled governance network.

##### 4.2.3.2 Analysis of application rules

From the perspective of application rules, policymakers have begun to emphasize the integration of industry and education, cooperation between medical schools, and the international development of MVE (51). From the perspective of evaluation criteria, employment rate, salary level, education quality, and social recognition have become important indicators for measuring the effectiveness of MVEPs. From the multi-level systems perspective, the government, schools, and medical institutions have formed a closer cooperation mechanism under the framework of high-quality development. At the operational selection level, policies have begun to focus on the internationalization, innovation, and radicalization of curriculum settings, teaching methods, and training bases, and to improve the international competitiveness and influence of MVE by introducing internationally advanced educational concepts and technical means. At the collective choice level, policymakers continue to explore new models for the deep integration of MVE and the medical industry. Innovations such as industry-education integration, medical school cooperation, and international cooperation aim to promote the deep integration of education and industry, thereby improving the pertinence and effectiveness of MVE. At the constitutional choice level, the government not only strengthens macro-guidance and supervision of MVE but also focuses on international integration and improving the global competitiveness and influence of MVE by strengthening international cooperation and exchanges. These efforts have jointly promoted the high-quality development of China’s MVE and provided strong talent support for economic and social development (25). At this point, the system of application rules exhibits characteristics of “system integration” and “cross-sector coupling.” It not only coordinates internal rules at various levels but also actively aligns with external international rules and industry innovation rules. This indicates that institutional development has entered a new stage of actively shaping and guiding community interaction and industrial development through rule innovation.

##### 4.2.3.3 Comprehensive analysis

This stage represents a paradigm shift from linear adjustment to networked coordination within the institutional system. First, the hyperdomainization of the institutional environment and the penetration of rules. The connection between the healthy China strategy and international medical standards forces the MVE system to seek a balance between “local characteristics” and “international alignment.” The subversion of medical education models by technologies such as AI and big data has reconstructed the “teaching-learning-evaluation” rule system. Then, the modernization and power flow of governance networks. The government has shifted from a “leader” to a “meta-governor” and promoted the coordination of multiple subjects by building a platform. The policy introduces corporate R&D resources and social capital into the MVE system through mechanisms such as the “Medical Education Innovation Consortium.” In short, this stage of transformation represents a paradigmatic shift within the IAD framework. The institutional system is shifting from adaptive feedback to internal and external variables, with a focus on strategic construction aimed at shaping the future. The introduction of the “high-quality development” goal is itself a powerful meta-rule, redefining the boundaries of the action arena, the roles of the participants, and their interaction patterns, thereby driving the entire policy system to evolve into a more complex and higher-level system.

### 4.3 Improvement of medical-vocational education policies in China

#### 4.3.1 Enhancing the adaptability of medical vocational education policies

Improving the adaptability of MVE has become a key entry point for promoting its high-quality development (74). To this end, it is necessary to start from the top-level design and optimize the focus of MVEPs in the new era to enhance the flexibility and adaptability of MVE. German MVE achieves flexible adjustment of teaching content through modular course design (75). Singapore’s “Skills Prosperity” program promotes technological change in the medical industry through real-time monitoring. Dynamic updates on skills certification standards for MVE (76). First, it is crucial to strengthen strategic awareness. We should closely combine the significant national development strategies, formulate a long-term development plan for MVE, and focus on responding to the diversified needs of high-quality development of MVE (77). Second, enhancing service awareness should not be ignored. Medical vocational education should actively respond to the new economic and social development needs and promote reform and innovation. Smooth the information exchange channels between “government, schools, enterprises, and society” and establish an effective cooperation mechanism. Promote the collaborative construction and sharing of resources from all parties to form a comprehensive and multi-level support system (78). In addition, humanistic awareness is also the key to improving the adaptability of MVE. We must focus on students, clarify the goals of talent training, and unswervingly implement the fundamental task of cultivating morality and people. In talent training, we must strengthen industry, academia, and research integration and pay equal attention to morality and ability. Finally, improving the training system, evaluation standards, and support mechanism of “dual-qualified” teachers is the basis for improving the quality of medical-vocational education. Teachers’ professionalism and practical ability directly affect students’ learning outcomes. In summary, the country can establish a “dynamic monitoring-rapid response” mechanism. A white paper on the matching degree between medical professional education programs and industry needs should be published every 2 years, providing quantitative analysis and early warnings of talent supply and demand gaps. Medical schools should be encouraged to establish a course module library based on real clinical cases to address sudden public health emergencies or regional medical technology upgrades. This aims to directly translate changes in the “natural attributes” (industry needs) within the IAD framework into subtle adjustments in “application rules” (course content) through institutionalized information channels and flexible regulations, thereby improving the system’s responsiveness.

#### 4.3.2 Optimizing the selection of policy tools for medical vocational education

Through the rational combination and flexible application of different policy tools, the effectiveness of policy implementation can be improved, the active participation of all parties can be promoted, and the comprehensive development of MVE can be promoted (79). Australia matches the financial subsidy direction through the “Skills Priority List” and provides an additional 30% funding support for training in scarce medical positions (80). First, the level and nature of policy goals should be clarified so that the corresponding tools can be reasonably selected. When facing clear and specific goals, command-type tools can be used first to ensure the rapid advancement of policies and the effective achievement of goals. Second, the choice of policy tools should also differ for different subjects (81). For multiple participants, including government, industry, enterprises, and social organizations, incentive tools such as financial subsidies and tax incentives can effectively stimulate their enthusiasm and initiative, promoting the effective allocation and use of resources. Finally, when selecting policy tools, it is necessary to make dynamic adjustments based on the actual situation of MVE development (82). With the development of the economy, society, and technological progress, the needs and challenges of MVE are constantly evolving. In short, it is recommended that a differentiated policy toolkit approach be implemented. To address the collective action dilemma at the operational level regarding the shortage of “dual-qualified” teachers, the government should utilize a combination of supply-side, environmental, and demand-side tools to address the bottlenecks at different levels of operational choices. For example, establishing a special fund for cultivating outstanding medical vocational education teachers and including the participation of clinical professionals from hospitals in teaching at vocational colleges is a necessary condition for their professional title evaluation.

#### 4.3.3 Implementing the responsibilities of key stakeholders in medical vocational education

In the modernization of medical-vocational education, clarifying the rights and responsibilities of each participant is a crucial foundation for the effective implementation of policies. To this end, it is necessary to reasonably define the duties and obligations of all parties at the three levels of government, industry, enterprises, and medical vocational schools to promote the overall development of MVE. The United Kingdom has established a “Health Education and Training Committee” across departments to coordinate resources from the Ministry of Health, Education, and the NHS. Data in 2022 show that this model has increased the matching rate of clinical internships for nursing students to 97% (83).

First, the central and local governments must clarify their dominant position in MVE. Still, they should not excessively interfere in the specific implementation to avoid the phenomenon of “overreaching.” The government’s primary responsibility is to formulate a clear policy framework and incentive mechanism to ensure the rational allocation of resources. Simultaneously, attention should be paid to the feedback from industry, enterprises, and medical vocational schools, and policies should be adjusted promptly to adapt to changing social needs. Second, as essential participants in MVE, industry enterprises are responsible for actively participating in curriculum design, internships, and medical education training. Enterprises should use their resources and experience to provide medical vocational schools with practice platforms and training opportunities to enhance students’ practical operation capabilities, especially clinical skills training. Concurrently, industry associations can play a bridging role in promoting communication and collaboration between the government, enterprises, and medical vocational schools (84). Finally, medical vocational schools should be primarily responsible for educating and cultivating medical talents. Colleges need to adjust their teaching content to meet the needs of the medical industry, actively cooperate with medical institutions, and carry out school-enterprise joint training projects to enhance students’ professional quality and employment competitiveness. Meanwhile, colleges must also establish and improve internal management systems and evaluation mechanisms to assess students’ comprehensive quality and skills, thereby achieving training goals (85). To promote the transition of responsibilities from “text” to “action” for all stakeholders, it is recommended that a “responsibility list-performance contract” management system be implemented. Provincial governments should collaborate with industry organizations to develop and publish a list of responsibilities and rights for the various stakeholders in medical vocational education. This list should clearly define the government’s fundamental responsibilities in terms of standards, funding, and evaluation, as well as the commitments of industry enterprises in curriculum development, job provision, and technology transfer.

## 5 Limitations

This study systematically analyzed China’s medical vocational education policies using text mining and the IAD framework, but it still has several limitations. First, the analytical perspective is limited to a macro-level approach. It primarily analyzes policy texts at the national level, failing to delve into the transmission and diffusion processes at the provincial and municipal levels. National policies may vary in their implementation at the local level due to differences in resources and contexts; their actual effectiveness and flexibility require empirical research based on local cases for evaluation. Second, the differences in policy types and implementation scenarios are not fully explored. While the overall themes of the policies were identified, the characteristics and differences in the design and implementation of policies at different levels (secondary vocational schools and higher vocational schools) and in various professional fields (such as nursing, medical technology, and rehabilitation therapy) were not systematically distinguished. The challenges and support required by various policies during implementation may be significantly specific. Third, the study focuses on the content analysis of static policy texts. While it can reveal the discourse focus and thematic shifts, it still falls short in tracking the dynamics of the actual policy implementation process, the real effects of resource allocation, and the long-term career development trajectories of graduates.

## 6 Conclusion and implications

### 6.1 Research conclusions

The research results show that: (1) The exogenous variables and natural attributes in the IAD framework are the main driving forces of changing MVEP. The weakening of China’s demographic dividend and the intensification of aging have led to a shift in the economy toward high-quality development, resulting in substantial industrial upgrading. The adjustment and transformation of the social economy directly affect the mainstream of MVE. Therefore, constantly adapting to the development needs of the medical industry and effectively serving the needs of economic construction are the basic requirements for the development of MVE. (2) The community attributes and interaction modes in the IAD framework provide ideas for sustainable MVEP development. The country actively promotes the integration of industry and education, as well as cooperation between medical schools in MVE, and actively integrates with international medical vocational education. This study offers a comprehensive theoretical analysis of the evolution and development of medical vocational education policies, providing a vital reference for policy formulation and practical application.

### 6.2 Research implications

From a theoretical perspective, this study validates and extends the applicability of the IAD framework in analyzing complex education policies. By combining objective econometrics of policy texts (such as cluster analysis and word frequency statistics) with the theoretical dimensions of institutional analysis, it provides an effective path to deconstruct the “black box” of medical vocational education policies and reveal their mechanisms of change. Future research could further integrate theories such as the Bayesian sponge framework, which focuses on information processing and cognitive evolution. This could analyze the dissemination, selection, and internalization of policy ideas among different actors, thus supplementing the “cognitive-behavioral” micro-explanatory dimension beyond “structure-institution” analysis.

From a practical perspective, policymaking should move beyond the current reliance on primarily “environmental” tools and strengthen the coordinated use of “supply-side” and “demand-side” tools. This will stimulate grassroots innovation and foster a sustainable, endogenous development mechanism. The government should promote balanced and integrated development, with a particular focus on the development of medical vocational education in the central and western regions.

Given the limitations of this study, future research could focus on the “local” aspects of policy implementation. Methods such as multi-case comparisons or policy ethnography could be employed to examine the transmission, translation, and implementation of the national MVEP (Multi-Minute Policy Implementation) across different regions and types of educational institutions. This would allow for the assessment of the policy’s actual adaptability, implementation deviations, and local innovation models. Secondly, a “categorized” and “differentiated” study of policies could be conducted. Comparing the commonalities and characteristics of policies at different levels (e.g., secondary vocational schools versus higher vocational schools) or in various professional fields in terms of goal setting, tool selection, and implementation effects would provide a basis for developing more professionally targeted support measures. Thirdly, a complementary theoretical framework could be introduced for micro-mechanism analysis. Theories focusing on information processing and cognitive evolution, such as the Bayesian sponge framework, could be integrated to provide a comprehensive understanding. Through questionnaires, interviews, or experiments, the study could reveal how policy actors receive, interpret, and internalize policy information. This would supplement the “structure-institution” explanation with the micro-mechanism of “cognition-behavior.”

## Data Availability

The datasets presented in this study can be found in online repositories. The names of the repository/repositories and accession number(s) can be found in the article/supplementary material.

## Footnotes

1 Source of data: http://www.moe.gov.cn/jyb_sjzl/sjzl_zcfg/zcfg_jyfl/202204/t20220421_620064.html

## References

[1] Li H Chen C Chen A Lin Q Li D Chen M . Effect of a three-years preventive medicine vocational education program on county-level healthcare workforce development in China: a cross-sectional study. BMC Med Educ. (2025) 25:522. doi: 10.1186/s12909-025-07095-w, 40217268 PMC11992889

[2] Feng S Li X Huang Z Jiang C Cheng X Ma Y . The relationship between burnout and sense of school belonging among the resident physicians in the standardization training in China. Med Educ Online. (2024) 29:2343515. doi: 10.1080/10872981.2024.2343515, 38660991 PMC11047212

[3] Yang L Chai Y Tang AM Wang XJ. Textual quantitative research and implications of China’s medical education policies-analysis based on the “tool-development-intensity” three-dimensional framework. BMC Med Educ. (2025) 25:1408. doi: 10.1186/s12909-025-08013-w, 41088331 PMC12522432

[4] Steinwachs DM Levine DM Elzinga DJ Salkever DS Parker RD Weisman CS. Changing patterns of graduate medical-education. N Engl J Med. (1982) 306:10–4. doi: 10.1056/NEJM198201073060103, 7053465

[5] Roberts C Conn JJ. Building capacity in medical education research in Australia. Med J Aust. (2009) 191:33–4. doi: 10.5694/j.1326-5377.2009.tb02672.x, 19580535

[6] Baig LA Ali SK Sarfaraz S. Role of politics, guilds and pedagogy in defining policies in medical education: the Pakistan scenario. Pak J Med Sci. (2022) 38:1708–13. doi: 10.12669/pjms.38.6.6057, 35991261 PMC9378366

[7] Le QC Bui MH Khuong QL Le PA Tran DT Nguyen Vu QH . Developing institutional policies for health professionals’ education reform: a case study of medical education in Viet Nam. Lancet Reg Health West Pac. (2025) 57:101551. doi: 10.1016/j.lanwpc.2025.101551, 40443537 PMC12121427

[8] Swails JL Angus S Barone MA Bienstock J Burk-Rafel J Roett MA . The undergraduate to graduate medical education transition as a systems problem: a root cause analysis. Acad Med. (2023) 98:180–7. doi: 10.1097/ACM.0000000000005065, 36538695

[9] Zhou YR Sun L Liang Y Mao G Xu P. Comprehensive quality of elderly rehabilitation nursing staff in medical and health care institutions in Liaoning province, China: a cross-sectional study. BMC Geriatr. (2022) 22:410. doi: 10.1186/s12877-022-03092-6, 35538424 PMC9087994

[10] Kaduszkiewicz H Teichert U van den Bussche H. Shortage of physicians in rural areas and in the public health service: a critical analysis of the evidence on the role of medical education and training. Bundes. Gesundheitsforschung-Gesundheitsschutz. (2018) 61:187–94. doi: 10.1007/s00103-017-2671-1, 29209761

[11] Savvidou E Evangelidis N Evangelidis P Avramidou E Nteli M Nteli D . Final-year medical students’ self-assessment of their competence to perform 123 clinical skills: a cross-sectional study in Greece. Hippokratia. (2024) 28:109–14.

[12] Pearson GME Ben-Shlomo Y Henderson EJ. A narrative overview of undergraduate geriatric medicine education worldwide. Eur Geriatr Med. (2024) 15:1533–40. doi: 10.1007/s41999-024-01055-1, 39317883 PMC11614947

[13] Wen SH Ren WM Qu L Wang Y Carline JD Fang GE. A survey on financial support and research achievement of medical education research units in China. Med Teach. (2011) 33:e158–62. doi: 10.3109/0142159X.2010.543442, 21345055

[14] Rominski S Donkor PA Lawson A Danso K Stern D. A low-cost method for performing a curriculum gap-analysis in developing countries: medical school competencies in Ghana. Teach Learn Med. (2012) 24:215–8. doi: 10.1080/10401334.2012.692266, 22775784

[15] Smit H Den Oudendammer F Kats E Van Lakerveld J. Lifelong learning on either side of the border: the effects of government policy on adult education in the Netherlands and Belgium. Eur J Educ. (2009) 44:257–70. doi: 10.1111/j.1465-3435.2009.01384.x

[16] Wang M Zheng Y Ma S Lu J. Does higher vocational education matter for rural revitalization? Evidence from China. Human Soc Sci Commun. (2024) 11:963. doi: 10.1057/s41599-024-03471-x

[17] Yang YJ Zheng J. Borrowing from Western countries? China’s vocational education, 1840–1895. Vocat Learn. (2025) 18:14. doi: 10.1007/s12186-025-09368-3

[18] Sun JJ Liu T Li H. Study on the status and problems of teaching system of “medical advanced mathematics”: data based on a research of 11 universities in China. BMC Med Educ. (2024) 24:36. doi: 10.1186/s12909-023-05012-7, 38191401 PMC10773098

[19] Xin Y Tang YB Mou XW. An empirical study on the evaluation and influencing factors of digital competence of Chinese teachers for TVET. PLoS One. (2024) 19:e0310187. doi: 10.1371/journal.pone.0310187, 39269950 PMC11398669

[20] Li YC Chen S Han B. A comparative analysis of orthodontic medical education and physician training models: China versus America and European countries. Eur J Dent Educ. (2025). doi: 10.1111/eje.70040, 40785140 PMC13383277

[21] Xiang K Liu J Chen X Zhang A. Research on the evolution and improvement of China’s shadow education governance policy from the perspective of multiple streams theory. Front Psychol. (2023) 14:1013243. doi: 10.3389/fpsyg.2023.1013243, 36993900 PMC10042137

[22] Ostrom E. Background on the institutional analysis and development framework. Policy Stud J. (2011) 39:7–27. doi: 10.1111/j.1541-0072.2010.00394.x

[23] Montes N Osman N Sierra C. A computational model of Ostrom’s institutional analysis and development framework. Artif Intell. (2022) 311:103756. doi: 10.1016/j.artint.2022.103756

[24] Biswas A. Developing a novel inclusive policy analysis framework based on capability approach and institutional analysis and development method. Soc Policy Adm. (2024) 59:101–18. doi: 10.1111/spol.13051

[25] Mcginnis MD. An introduction to IAD and the language of the Ostrom workshop: a simple guide to a complex framework. Policy Stud J. (2011) 39:169–83. doi: 10.1111/j.1541-0072.2010.00401.x

[26] Ostrom E. An agenda for the study of institutions. Public Choice. (1986) 48:3–25. doi: 10.1007/BF00239556

[27] Barak MH Shoshana A. ‘Soon you will go far’: discourse analysis on vocational education policy of Israel. Compare J Comp Int Educ. (2024) 55:887–905. doi: 10.1080/03057925.2024.2321886

[28] Barabasch A Watt-Malcolm B. Teacher preparation for vocational education and training in Germany: a potential model for Canada? Compare J Comp Int Educ. (2013) 43:155–83. doi: 10.1080/03057925.2012.661216

[29] Klatt M Polesel J. Vocational education and training in Australia and three-dimensional federalism. Aust J Educ. (2013) 57:74–86. doi: 10.1177/0004944112468702

[30] Hasanefendic S Heitor M Horta H. Training students for new jobs: the role of technical and vocational higher education and implications for science policy in Portugal. Technol Forecast Soc Chang. (2016) 113:328–40. doi: 10.1016/j.techfore.2015.12.005

[31] Choi SJ Jeong JC Kim SN. Impact of vocational education and training on adult skills and employment: an applied multilevel analysis. Int J Educ Dev. (2019) 66:129–38. doi: 10.1016/j.ijedudev.2018.09.007

[32] Brunetti I Corsini L. School-to-work transition and vocational education: a comparison across Europe. Int J Manpow. (2019) 40:1411–37. doi: 10.1108/IJM-02-2018-0061

[33] Chen J Pastore F. Dynamics of returns to vocational education in China: 2010–2017. Human Soc Sci Commun. (2024) 11:118. doi: 10.1057/s41599-024-02616-2

[34] Yi HM Yi H Zhang L Liu C Chu J Loyalka P . How are secondary vocational schools in China measuring up to government benchmarks? China World Econ. (2013) 21:98–120. doi: 10.1111/j.1749-124x.2013.12024.x

[35] Ye R Chudnovskaya M Nylander E. Right competence at the right time-but for whom? Social recruitment of participants in an expanding higher vocational education segment in Sweden (2005–2019). Adult Educ Q. (2022) 72:380–400. doi: 10.1177/07417136221080423

[36] Allais S. Will skills save us? Rethinking the relationships between vocational education, skills development policies, and social policy in South Africa. Int J Educ Dev. (2012) 32:632–42. doi: 10.1016/j.ijedudev.2012.01.001

[37] Maurer M. Recognizing prior learning in vocational education and training: global ambitions and actual implementation in four countries. Comp Educ. (2023) 59:1–17. doi: 10.1080/03050068.2022.2132445

[38] Yang J. General or vocational? The tough choice in the Chinese education policy. Int J Educ Dev. (1998) 18:289–304. doi: 10.1016/S0738-0593(98)00026-1

[39] Williams S. Policy tensions in vocational education and training for young people: the origins of general national vocational qualifications. J Educ Policy. (1999) 14:151–66. doi: 10.1080/026809399286422

[40] Maliranta M Nurmi S Virtanen H. Resources in vocational education and post-schooling outcomes. Int J Manpow. (2010) 31:520–44. doi: 10.1108/01437721011066344

[41] Lee SY Lee JCK Lam BYH. Does renaming improve public attitudes toward vocational education and training in higher education? Evidence from a survey experiment. Educ Train. (2022) 64:347–59. doi: 10.1108/ET-01-2021-0014

[42] Ling Y Jeong SJ Wang LW. Research on the reform of management system of higher vocational education in China based on personality standard. Curr Psychol. (2023) 42:1225–37. doi: 10.1007/s12144-021-01480-6

[43] Xue EY Li J. Exploring the macro education policy design on vocational education system for new generation of migrant workers in China. Educ Philos Theory. (2020) 52:1028–39. doi: 10.1080/00131857.2019.1675468

[44] Cheng C Cheng S Feng CS. The triple helix model for industry-education city integration in China: a development approach. SAGE Open. (2024) 14:21582440241. doi: 10.1177/21582440241250111

[45] Hardy I Liu SY. Complex connectivities: policy networks, data infrastructures and vocational education reform in China. Int J Educ Res. (2022) 115:102045. doi: 10.1016/j.ijer.2022.102045

[46] Salajan FD Roumell EA. Tracing the historical construction of a vocational training, adult education and lifelong learning policy space in the European Union from 1951 to present. Eur Educ Res J. (2023) 22:347–67. doi: 10.1177/14749041211065324

[47] Gu JF. Spatial diffusion of social policy in China: spatial convergence and neighborhood interaction of vocational education. Appl Spat Anal Policy. (2016) 9:503–27. doi: 10.1007/s12061-015-9161-3

[48] Heikkila T Andersson K. Policy design and the added-value of the institutional analysis development framework. Policy Polit. (2018) 46:309–24. doi: 10.1332/030557318X15230060131727

[49] McMullin C Roy MJ Curtin M. Institutional logics as a framework for understanding third sector development: an analysis of Quebec and Scotland. Policy Polit. (2021) 49:615–32. doi: 10.1332/030557321X16239357875918

[50] Hardy SD Koontz TM. Rules for collaboration: institutional analysis of group membership and levels of action in watershed partnerships. Policy Stud J. (2009) 37:393–414. doi: 10.1111/j.1541-0072.2009.00320.x

[51] Radaelli CM Rangoni B. Regulation and its metrics: three views of the cathedral. J Eur Publ Policy. (2025). doi: 10.1080/13501763.2025.2501085

[52] Li YZ Liu J Yang H Chen J Xiong JJ. A bibliometric analysis of literature on vegetable prices at domestic and international markets-a knowledge graph approach. Agriculture. (2021) 11:951. doi: 10.3390/agriculture11100951

[53] Crowther D Kim S Lee J Lim J Loewen S. Methodological synthesis of cluster analysis in second language research. Lang Learn. (2021) 71:99–130. doi: 10.1111/lang.12428

[54] Jain AK Murty MN Flynn PJ. Data clustering: a review. ACM Comput Survey. (1999) 31:264–323. doi: 10.1145/331499.331504

[55] Liu Y Li BF. Bayesian hierarchical *K*-means clustering. Intell Data Anal. (2020) 24:977–92. doi: 10.3233/IDA-194807

[56] Yun DJ Jung H Kang H Yang WY Seo DW. Acceleration of the multi-level fast multipole algorithm using K-means clustering. Electronics. (2020) 9:1926. doi: 10.3390/electronics9111926

[57] Riaz S Fatima M Kamran M Nisar MW. Opinion mining on large scale data using sentiment analysis and k-means clustering. Cluster Comput. (2019) 22:7149–S7164. doi: 10.1007/s10586-017-1077-z

[58] Kuo RJ Wang HS Hu TL Chou SH. Application of ant K-means on clustering analysis. Comput Math Appl. (2005) 50:1709–24. doi: 10.1016/j.camwa.2005.05.009

[59] Vuong Q-H Nguyen M-H La V-P. The Mindsponge and BMF Analytics for Innovative Thinking in Social Sciences and Humanities. Berlin: De Gruyter (2022).

[60] Sari NPWP Mazenda A Katiyatiya CLF Nguyen MH Vuong QH. Policy analysis in school meals program: regulation impacts on in-school food fortification. J Hunger Environ Nutr. (2025) 20:1024–41. doi: 10.1080/19320248.2025.2505041

[61] Stewart LA Clarke M Rovers M Riley RD Simmonds M Stewart G . Preferred reporting items for a systematic review and meta-analysis of individual participant data the PRISMA-IPD statement. JAMA. (2015) 313:1657–65. doi: 10.1001/jama.2015.3656 25919529

[62] Vuong QH La VP Nguyen MH. Informational entropy-based value formation: a new paradigm for a deeper understanding of value. Eval Rev. (2025). doi: 10.1177/0193841X251396210, 41214459

[63] Rodriguez-Rodriguez FJ Hierro LA Garzon AJ. Fed and ECB reaction functions during quantitative easing: three phases of monetary policy, both conventional and unconventional. J Policy Model. (2024) 46:928–45. doi: 10.1016/j.jpolmod.2024.03.003

[64] Amiel M Yemini M. Who takes initiative? The rise of education policy networks and the shifting balance of initiative-taking amongst education stakeholders in Israel. J Educ Policy. (2023) 38:586–606. doi: 10.1080/02680939.2022.2130996

[65] Baizan P Nie WL. The Impact of education on fertility during the Chinese reform era (1980–2018): changes across birth cohorts and interaction with fertility policies. Eur J Popul. (2024) 40:7. doi: 10.1007/s10680-023-09691-2 38289489 PMC10828303

[66] Mauger P. Changing policy and practice in Chinese rural education. China Q. (1983) 93:138–48. doi: 10.1017/S0305741000016210

[67] Lv Y Wu M Shouse RC. Impact of organizational culture, occupational commitment and industry-academy cooperation on vocational education in China: cross-sectional hierarchical linear modeling analysis. PLoS One. (2022) 17:e0264345. doi: 10.1371/journal.pone.0264345, 35196361 PMC8865651

[68] Zhang HY Shi YY. Evolution of English language education policies in the Chinese mainland in the 21st century: a corpus-based analysis of official language policy documents. Linguist Educ. (2023) 76:101190. doi: 10.1016/j.linged.2023.101190

[69] Xu W. Learning to ‘tell China’s story well’: higher education policy and public diplomacy in Chinese international education. J High Educ Policy Manag. (2024) 46:166–81. doi: 10.1080/1360080X.2023.2269499

[70] Jin X Zhang C Su J. The current situation and problems of major offerings in higher vocational colleges based on industry 4.0: a case study of higher vocational colleges in Xiamen. Mob Inf Syst. (2022) 2022:5618247. doi: 10.1155/2022/5618247

[71] Tschopp M Bieri S Rist S. Quinoa and production rules: how are cooperatives contributing to governance of natural resources? Int J Commons. (2018) 12:402–27. doi: 10.18352/ijc.826

[72] Khan MS Shoaib A Arledge E. How to promote AI in the US federal government: insights from policy process frameworks. Gov Inf Q. (2024) 41:101908. doi: 10.1016/j.giq.2023.101908

[73] Srigiri SR Dombrowsky I. Analysing the water-energy-food Nexus from a polycentric governance perspective: conceptual and methodological framework. Front Environ Sci. (2022) 10:725116. doi: 10.3389/fenvs.2022.725116

[74] Hou CX Zhou L Wen Y Chen Y. Farmers’ adaptability to the policy of ecological protection in China-a case study in Yanchi county, China. Soc Sci J. (2018) 55:404–12. doi: 10.1016/j.soscij.2018.06.001

[75] Sternberg A Fauser D Banaschak H Bethge M. Sequences of vocational rehabilitation services in Germany: a cohort study. BMC Health Serv Res. (2024) 24:74. doi: 10.1186/s12913-023-10499-3, 38225557 PMC10788977

[76] Wong TY Cheong SK Koh GCH Goh LG. Translating the family medicine vision into educational programmes in Singapore. Ann Acad Med Singap. (2008) 37:421–5. doi: 10.47102/annals-acadmedsg.V37N5p421, 18536831

[77] Gills R Ramachandran C Vipinkumar VP Kumar M Varghese E Jayasankar J . Education-world of work mismatch: a multidimensional competence gap analysis for reorienting the fisheries vocational education system in India. Curr Sci. (2023) 124:1329–38. doi: 10.18520/cs/v124/i11/1329-1338

[78] Carstensen MB Ibsen CL Jensen IMN. Integrating ecosocial policies through polycentric governance: a study of the green transformation of Danish vocational education and training. Regulat Govern. (2024). doi: 10.1111/rego.12633

[79] Tian HW Cui Y Dong J. Research on policy instruments for promoting green lifestyle in China-a multi-dimensional analysis based on current policy texts. Front Environ Sci. (2024) 12:1405537. doi: 10.3389/fenvs.2024.1405537

[80] Russell DJ Monani D Martin P Wakerman J. Addressing the GP vocational training crisis in remote Australia: lessons from the northern territory. Aust J Rural Health. (2023) 31:967–78. doi: 10.1111/ajr.13029, 37607122

[81] Wu X Wang M. Selection of cooperative enterprises in vocational education based on ANP. Educ Sci Theory Prac. (2018) 18:1507–15. doi: 10.12738/estp.2018.5.047

[82] Köpsén J. Demands-based and employer-driven curricula: defining knowledge in higher vocational education and training. Stud Contin Educ. (2020) 42:349–64. doi: 10.1080/0158037X.2019.1661238

[83] Hays RB. Reforming medical education in the United Kingdom: lessons for Australia and New Zealand. Med J Aust. (2007) 187:400–3. doi: 10.5694/j.1326-5377.2007.tb01312.x, 17908005

[84] Setó-Pamies D Domingo-Vernis M Rabassa-Figueras N. Corporate social responsibility in management education: current status in Spanish universities. J Manag Organ. (2011) 17:604–20. doi: 10.5172/jmo.2011.17.5.604

[85] Abrahaoa VD Vaquero-Diegoa M Móstolesb RC. University social responsibility: the role of teachers. J Innov Knowl. (2024) 9:100464. doi: 10.1016/j.jik.2024.100464

